# The monoclonal antibody SM5-1 recognizes a fibronectin variant which is widely expressed in melanoma

**DOI:** 10.1186/1471-2407-6-8

**Published:** 2006-01-11

**Authors:** Uwe Trefzer, Yingwen Chen, Gunda Herberth, Maja Ann Hofmann, Felix Kiecker, Yajun Guo, Wolfram Sterry

**Affiliations:** 1 Department of Dermatology and Allergy, Skin Cancer Center, Charité – Universitätsmedizin Berlin, Schumannstrasse 20/21, 10117 Berlin, Germany; 2 Centre for Environmental Research Leipzig – Halle Ltd., Department of Environmental Immunology, Leipzig, Germany; 3 International Cancer Institute and Eastern Institute of Hepatobiliary Surgery, the Second Military Medical University, Shanghai, China

## Abstract

**Background:**

Previously we have generated the monoclonal antibody SM5-1 by using a subtractive immunization protocol of human melanoma. This antibody exhibits a high sensitivity for primary melanomas of 99% (248/250 tested) and for metastatic melanoma of 96% (146/151 tested) in paraffin embedded sections. This reactivity is superior to the one obtained by HMB-45, anti-MelanA or anti-Tyrosinase and is comparable to anti-S100. However, as compared to anti-S100, the antibody SM5-1 is highly specific for melanocytic lesions since 40 different neoplasms were found to be negative for SM5-1 by immunohistochemistry. The antigen recognized by SM5-1 is unknown.

**Methods:**

In order to characterize the antigen recognized by mAb SM5-1, a cDNA library was constructed from the metastatic human melanoma cell line SMMUpos in the Uni-ZAP lambda phage and screened by mAb SM5-1. The cDNA clones identified by this approach were then sequenced and subsequently analyzed.

**Results:**

Sequence analysis of nine independent overlapping clones (length 3100–5600 bp) represent fibronectin cDNA including the ED-A, but not the ED-B region which are produced by alternative splicing. The 89aa splicing variant of the IIICS region was found in 8/9 clones and the 120aa splicing variant in 1/9 clones, both of which are included in the CS1 region of fibronectin being involved in melanoma cell adhesion and spreading.

**Conclusion:**

The molecule recognized by SM5-1 is a melanoma associated FN variant expressed by virtually all primary and metastatic melanomas and may play an important role in melanoma formation and progression. This antibody is therefore not only of value in immunohistochemistry, but potentially also for diagnostic imaging and immunotherapy.

## Background

Melanoma-associated antigens such as MelanA/Mart-1 or tyrosinase recognized by monoclonal antibodies can be used as diagnostic markers for immunohistochemistry or as therapeutic targets for specific immunotherapy. Previously we have produced a panel of monoclonal antibodies (mAb) by subtractive immunization of the human melanoma cell line SMMUneg, generated from a primary melanoma and the SMMUpos cell line, generated from the same patient's metastatic melanoma [1]. One of the antibodies, mAb SM5-1 was found to react with SMMUpos, but not with SMMUneg, being suggestive for the recognition of a metastases associated molecule. Upon detailed screening we found that SM5-1 and HMB-45 had a comparable sensitivity of 97% to 99% in detecting paraffin embedded primary melanomas, but SM5-1 was superior to HMB-45 in detecting metastases (146/151, 96% vs. 126/151, 83%). SM5-1 was shown to be highly specific for melanocytic lesions with negative staining of 40 different non-melanocytic neoplasms [2]. Moreover, when we compared the immunohistochemical staining pattern of SM5-1 with that of anti-MART-1 (mAb A103) and anti-tyrosinase (mAb T311) we found an overall reactivity of 92% (318/344) for SM5-1, 83% (285/344) for MART-1 and 71% (245/344) for tyrosinase in primary and metastatic melanoma specimens. 52 of 56 MART-1-negative and 81 of 89 tyrosinase-negative metastases were positive for SM5-1 [3]. Therefore, mAb SM5-1 is of high value in immunohistochemistry of melanoma, though the antigen recognized by SM5-1 is unknown. The differential screening of a cDNA library constructed from metastatic and non-metastatic variants is used to identify novel genes possibly associated with the progress of melanoma [4,5]. Some genes identified by cDNA screening were either higher or lower expressed in the metastatic counterpart than in the primary one [6]. In the presented study, a cDNA library of SMMUpos cells was constructed into the Uni-ZAP lambda phage system and screened with SM5-1 in order to identify the antigen recognized by SM5-1.

## Methods

### Cell lines and antibodies

The human melanoma cell lines SMMUpos and SMMUneg were established from the primary and metastatic lesion, respectively, of the same patient and were cultured in RPMI 1640 (GibcoBRL), supplemented with 10% FCS (GibcoBRL), 2 mM L-Glutamine (GibcoBRL), 50 I.U./ml penicillin (Sigma) and 50 ug/ml streptomycin (Sigma) at 37°C, 5% CO_2 _in a humidified incubator. The 47 other melanoma cell lines were generated from metastatic lesions of melanoma patients and were treated and kept identical as the SMMU cell lines. Informed consent was obtained from all patients prior to tissue sampling. The mAb SM5-1 (mouse IgG1) was generated as previously described. The Fibronectin mAbs TV-1 (Dianova, Hamburg, Germany) and FN-15 (ICN Biomedicals, OH, USA) are both mouse IgG1 antibodies and were used at a dilution of 1:100 or 1:200, respectively. FITC-conjugated goat anti-mouse IgG1 (Dianova) was used as controls at 1:10.

### Flow cytometry and cytospins

The cell monolayer were harvested by a brief exposure to 0.05% trypsin/0.02% EDTA in PBS (GibcoBRL), resuspended in PBS and washed twice and resuspended in the staining buffer (10% inactivated FCS/0.1% sodium azide in PBS, Sigma) containing 10 ug/ml of mAb SM5-1 or mouse IgG1 (Sigma) as negative control. After 30 minutes at 4°C incubation, cells were washed and resuspended in the staining buffer containing FITC labeled goat anti-mouse IgG1, incubated for 30 minutes at 4°C and analyzed with a flow cytometer (FacsCalibur, Becton Dickinson). For staining cytospins the cells were washed and suspended on a glass microscope slide, followed by centrifugation for 4 minutes at 400 rpm in a Shandon Cytospin 2 (Shandon, Germany). The slides were fixed in acetone and stained with a two antibodies/streptavidin-biotin (LSAB) method (DAKO). Slides were incubated with 10 ug/ml SM5-1 antibody or mouse IgG1, washed, incubated with a biotinylated link antibody (DAKO) and peroxidase-labeled streptavidin (DAKO). Colour was developed by using Diaminobenzidine as chromogen.

### mRNA extraction and cDNA library construction

Cultured SMMUpos cells (4 × 10^7^) were incubated with denaturing solution, incubated on ice for 5–10 minutes and centrifuged at 15,000 × g for 5 minutes at 4°C. After two rounds of phenol:chloroform extractions 10.0 ml isopropanol (Sigma) was added, mixed, incubated on ice for 10 minutes and washed. 0.5 ug total RNA, 100 ul 10× Dnase buffer I and 5 ul Dnase I (10 units/ul) (Boehringer) were brought to a volume of 1 ml by adding deionized Rnase-free water. The reaction mix was incubated at 37°C for 1 hour, followed by addition of 100 ul 10 × Termination Mix (1.0 M EDTA, ph 8.0; 1.0 mg/ml Glycogen (Sigma). 1 volume phenol:chloroform: isoamyl alcohol (25:24:1) (Biorad) was added, vortexed vigorously and centrifuged at 12,000 × g for 15 minutes at 4°C. 100 ul of 7.5 M NH_4_OAC and 1.5 ml 96% ethanol were added, vortexed thoroughly and spun at 12,000 × g for 20 minutes. 12.5 mM Tris-HCL (pH 8.3), 18.75 mM KCL, 0.75 mM MgCl_2_, 5 mM dNTP (GibcoBRL) were added to 5 ug total RNA and incubated at 42°C for 2 minutes. 200 units SUPERSCRIPT II (GibcoBRL) were added, incubated for further 50 minutes, followed by 70°C for 15 minutes. The cDNA was unidirectionally inserted into the Uni-ZAP™XR vector system (Stratagene, Ja Jolla, CA), which is known for the high efficiency of lambda library construction and the convenience of white-blue color selection. The inserts can be in-vivo excised in form of the pBluescript SK(-) phagemid. The library was synthesized using the ZAP-cDNA^® ^synthesis method [7]. The library was aliquoted, DMSO was added to a final concentration of 7% and stored at -80°C.

### Immunoscreening of cDNA library

Amplification and screening of the cDNA were performed in E. coli XL1-blue host strain. The bacterial cells were coincubated with the phage and incubated for 15 minutes at 37°C to allow the phage to absorb to the bacterial cells. Top agar was added to the mixture, poured onto agar plates, distributed and incubated at 42°C until small plaques became visible. Nitrocellulose membranes were submersed with 10 mM IPTG, dried, applied to the agar plates and incubated for 3.5 hours at 37°C. The membranes were washed in TBST, immersed in blocking solution and incubated with 8 ug/30 ml blocking solution mAb SM5-1. After washing 0.2 ug/ml Ab-AP conjugate was added for 1 hour at RT. The membranes were then immersed in the BCIP (0.3 mg/ml)-NBT (0.15 mg/ml) (Stratagene) color development solution until positive reactions were clearly seen. Membranes were aligned and positive clones plugged from the agar, transferred to 1 ml of SM buffer with 1 drop of chloroform and incubated at RT for 1–2 hours. After centrifugation the supernatant was collected and chloroform added to a final concentration of 0.3%. XL-1 Blue cells, diluted in 10 mM MgSO_4 _to an OD_600 _= 0.5 were mixed with 300 ul of the picked clones, incubated for 15 minutes at 37°C, mixed with melted top agar and plated on 150-mm plates of bottom agar. After 6–8 hours incubation the agar-plates were overlaid with 8–10 ml of SM buffer and stored at 4°C with shaking. On day three the phage suspension was removed and chloroform added to a final concentration of 5%, incubated at RT and washed.

### In vivo excision using the ExAssist/SOLR system

The excision of the cloned fragments was performed according to the manufacturer's manual (Stratagene). XL1-Blue cells were co-infected with the phage clones (containing >1 × 10^5 ^phage particles) and helper phage ExAssist (>1 × 10^6 ^pfu/ml) for 15 min at 37°C, followed by incubation in LB broth for 2–2.5 hours at 37°C with shaking. The supernatant was heated at 70°C for 15 minutes to kill the host cells and release the phagemids. The cell-phage mixture was centrifuged for 15 minutes at 4000 × g and the supernatant containing phagemids was incubated with E. coli SOLR (OD_600 _= 1.0) cells to produce plasmid clones and plated on LB-ampicillin plates for overnight incubation at 37°C.

### DNA sequencing

For automated DNA sequencing (Model 375 DNA sequence system, APPLIED BIOSYSTEMS) the plasmid DNA was purified using the QIAprep plasmid preparation system (QIAgen, Hilden, Germany). The primers used in the sequencing reaction are listed in Additional file 1. 10 ul product of each sequencing reaction was digested with 10 U EcoR I and 10 U Xho I and run on a 0.8% agarose gel. The PCR products were mixed with 2 ul 3 M NaAC ph 5.4 and 50 ul 95% ethanol, incubated on ice for 10 minutes followed by centrifugation at 14,000 rpm for 30 minutes. The pellets were washed with 250 ul of 70% ice cold ethanol, centrifuged at 14,000 rpm for 10 minutes, air dried and resuspended with 4 ul loading solution (5:1:1 deionized formamide: 50 mM EDTA: Dextran blue), heated at 90°C for 2 min, cooled and run on the sequencing gel at 1600 V, 25 mA, 30 W for at least 10 hours. The sequencing results were compared with Genbank Entries (BLAST).

### RT-PCR

The following primers were used: human beta actin (accession number: X00351): 5' primer: AAC CTA ACT TGC GCA GAA AAC (1209 bp to 1230 bp), 3'primer: TTT ACA CGA AAG AAC TGC TAT C (1551 bp to 1530 bp); human fibronectin (accession number: X02761): 5'primer: CTG TTA CTG GTT ACA GAG TAA (4623 bp to 4643 bp), 3'primer: TAG GTC ACC CTG TAC CTG GAA (4917 bp to 4897 bp). Each PCR reaction was performed in 10 mM Tris-HCL, ph 8.3, 50 mM KCL, 3 mM MgCl_2_, 0,2 mM dNTP, 0.4 uM 3'primer, 0.4 um 5'primer, 2.5 units AmpliTaq Gold and 1 ul of the cDNA, prepared by reverse transcription. The PCR program used was: 10 min at 94°C, 26 cycles of (30 s at 94°C, 1 min at 51°C, 30 s at 72°C), followed by a final additional extension at 72°C for 5 min. The products were electrophoresed on a 1.0% agarose gel.

### Western blotting

Cell suspensions of SMMUpos or SMMUneg cells were lysed in lysis buffer and 50 μg of protein was electrophoresed on a 5% polyacrylamide gel in a minigel apparatus (Bio-Rad), the gel was electrotransferred to nitrocellulose membranes (Schleicher & Schüll), washed with distilled water for 5 min and blocked for 1 hour using a blocking solution composed of 2% milk powder in PBS. After 3 × 10-min washes in PBS/0.05% Tween 20 (Sigma), mAb SM5-1 (0.1 ug/ml) was incubated with the membrane in blocking solution for 60 min, washed and incubated with a secondary Ab (peroxidase-conjugated goat anti-mouse IgG1) (Dianova) at a 1/250 dilution in blocking solution for one hour. After further 3 × 10-min washes, the membrane was incubated with the ECL reagent for 2 min and exposed to x-ray film for 90 s. A broad-range protein standard marker was used for size determination.

## Results

### mAb SM 5-1 is reactive with SMMUpos cells and other melanoma cell lines, but not with SMMUneg cells

**Figure 1:**
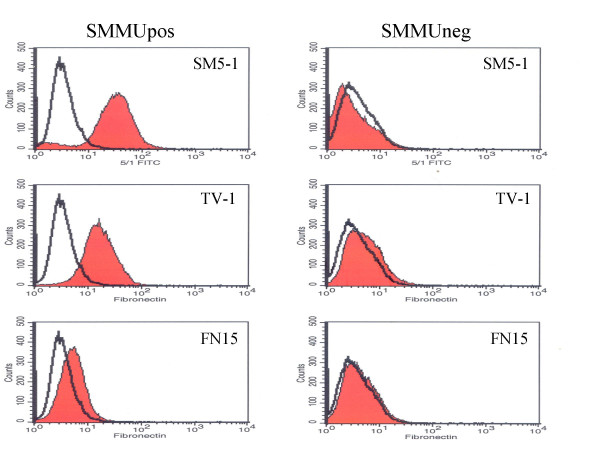
**FACS analyses with SM5-1 and Fibronectin antibodies**. SMMUpos cells react with mAb SM5-1 and the fibronectin mAbs TV-15 and FN15 while SMMUneg cells are negative for all mAbs.

**Figure 2:**
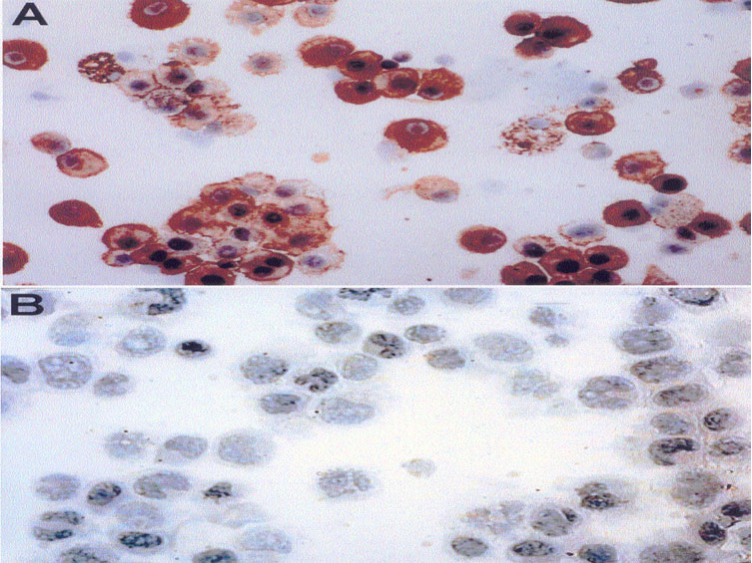
**Cytospins with mAb SM5-1**. Immunohistochemical staining (cytospin) of SMMUpos (A) or SMMUneg (B) cells with mAb SM5-1.

**Figure 3:**
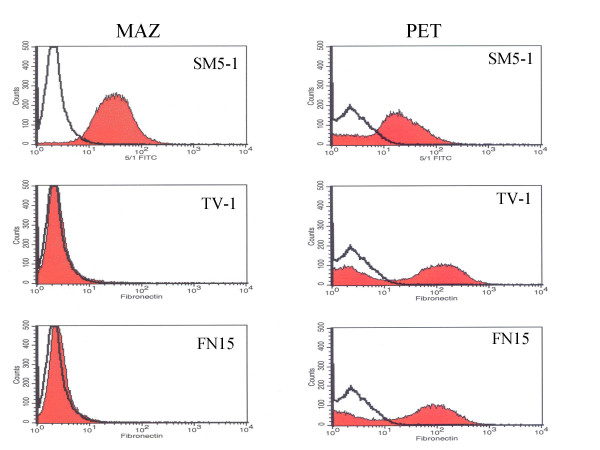
**FACS analyses with SM5-1 and Fibronectin antibodies in MAZ and PET cell line**. Human melanoma cell lines MAZ and PET react with mAb SM5-1. MAZ reacts also with fibronectin mAbs TV-15 and FN15 while PET does not.

**Figure 4:**
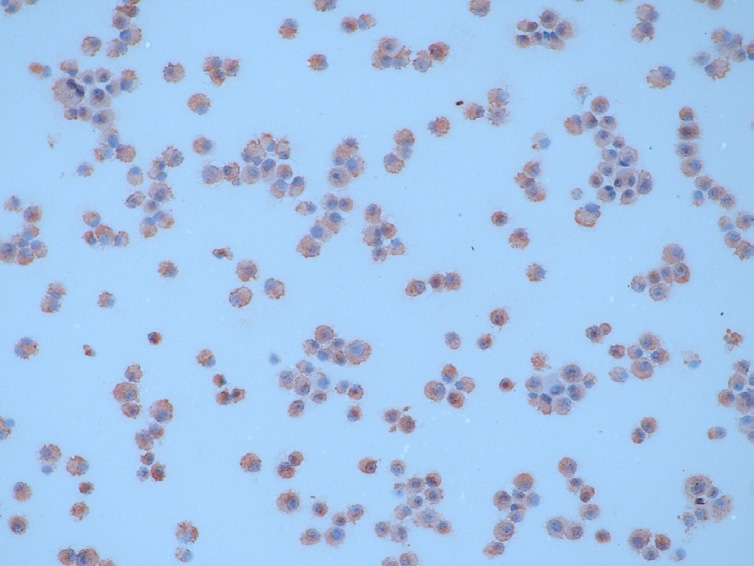
**Cytospin of cell line MAZ immunostained with mAb SM5-1**. Immunohistochemical staining (cytospin) of MAZ cells with mAb SM5-1.

**Figure 5:**
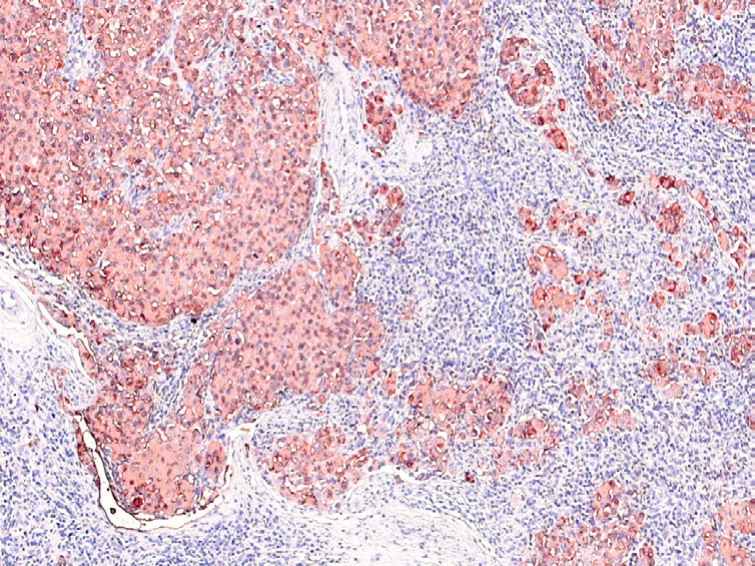
**Lymph node metastasis of patient MAZ immunostained with mAb SM5-1**. Immunohistochemical staining of a lymph node metastasis of patient MAZ.

The mAb SM5-1 was generated by subtractive immunization of mice with the human primary melanoma cell line SMMUneg and the human metastatic melanoma cell line SMMUpos established from the same patient. Theoretically, the antigen recognized by SM5-1 should be associated with the metastatic behavior of the melanoma and may also only be expressed in metastatic melanoma cell SMMUpos, but not in primary melanoma cell SMMUneg. As shown in Fig. 1, upper part (FACS analyses) and Fig. 2 (cytospins), the SMMUpos cell line is strongly positive for SM5-1, while SMMUneg is not. Therefore, SM5-1 recognizes an antigen which is differentially expressed by the two cell lines. In order to confirm the reactivity of SM5-1 with other human melanoma tissue, 47 different human melanoma cell lines were immunostained and analyzed by FACS. All 47 cell lines were positive for SM5-1. Examples are shown in Fig. 3, upper part for the cell lines MAZ and PET as well as for the SM5-1 stained cytospin (Fig. 4) or lymph node metastasis (Fig. 5) of Patient MAZ. SMMU pos cells also reacted with the two fibronectin antibodies TV-1 and FN15 while SMMUneg cells were completely negative for these antibodies, suggesting a complete loss of fibronectin in SMMUneg and further suggesting, that mAb SM5-1 might recognize the same pan-fibronectin form as TV-1 and FN15 (Fig. 1). TV-1 and FN15 were reactive in only a subset of the 47 melanoma cell lines. One example is shown in Fig. 3 with absent and present reactivity for the TV-1 and FN15 antibodies in the melanoma cell line MAZ and PET, respectively.

### Immunoscreening of SMMUpos human melanoma cDNA library with mAb SM5-1

After the initial screening of independent recombinants, 192 putative clones were identified. These clones were taken through several rounds of purification by replating and rescreening with mAb SM5-1. After further rounds of screening, nine clones (clones 127-1a, 130-1, 131-2-2, 139-1, 146-1, 181-2, 184-1, 185, 187-1) recognized by mAb SM5-1 were confirmed. Several positive plaques from each clone were picked and re-plated out in a 1:100 dilution of the plasmid (about 30–50 plaques in one 82 mm plate). A single positive clone was further picked and reamplified by plating out in high concentration on LB-tetracycline plates. At this point all plaques gave an equally strong signal and all clones were considered pure positive ones.

### Sequence of the full length of the positive clones

**Figure 6:**
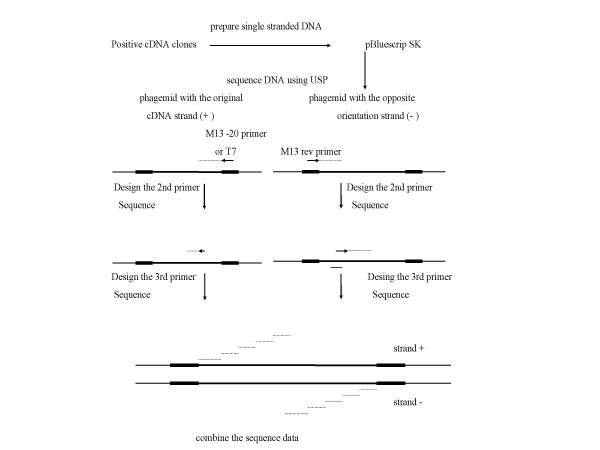
**Sequencing strategy of positively identified clones**. The insert was first sequenced by the universal primer T7 (or M13 -20 primer) in one orientation and by the universal M13 reverse primer in the reverse orientation. Since the range of sequenced DNA was short (a few hundred base pairs), a sequence from near the end of this run was used to design a second primer, which primed the sequence from this region and moved another few hundred nucleotides further. From the end of this run the third primer was designed and the circle was repeated until the entire insert had been covered.

All nine positive clones were completely sequenced (Fig. 6, sequencing strategy) and compared with the Genbank sequences (BLAST at NCBI). Each primer was designed based on three criteria: 1) primer length should be 20 bps 2) the GC content should be approximately 50% of the primer sequence 3) the 3' primer sequence should not be AT, TA, GC or CG. Two panels of primers were designed, one for the original strand and one for the reverse strand. In order to exclude mutations in the sequence, the sequencing was repeated three times at the same region and the sequenced regions completely overlapped. The primers (listed in Additional file 1) and the locations of the primers are shown in the Additional file 2 in bold and italic sequence. The sequence results showed that all nine clones were similar to the human cellular fibronectin mRNA (Accession Number: X02761) and the identity scores were about 98%. The deduced amino acid sequences of the positive clones were 100% identical to the human fibronectin sequence. The sequence of the positive clones compared with fibronectin pre-mRNA is shown in Additional file 2. All nine clones overlapped. The shortest clone, which spanned the segment of fibronectin from 4503 bp to the poly(A) end, was sequenced four times in order to be certain that there was no mutation in this region. The shortest clone included the ED-A region, but excluded the RGD motif. There was no ED-B region in any of the clones.

### Restriction map of positive cDNA clones

**Figure 7:**
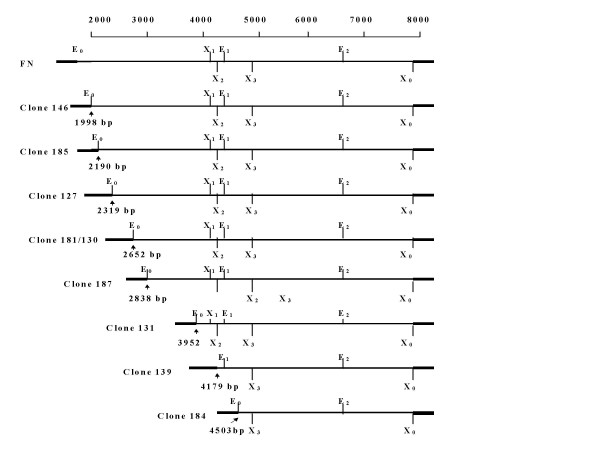
**Alignment of nine positive clones' fragments**. Alignment of nine positive clones' fragments from EcoR I and Xho I digestion and comparison to fibronectin. E_0_: EcoR I site of polylinker; E_1_: EcoR I site in fibronectin DNA sequence at position 4150; E_2_: EcoR I site in fibronectin DNA sequence at position 6680; X_0_: Xho I site of polylinker; X_1_: Xho I site in fibronectin DNA sequence at position 4062; X_2_: Xho I site in fibronectin DNA sequence at position 4122; X_3_: Xho I site in fibronectin DNA sequence at position 4918. All nine clones overlapped from the poly(A). The shortest clone 184 spanned the sequence from the poly(A) end to 4503 bp of fibronectin; the longest clone146 spanned from poly(A) end to 1998 bp of fibronectin.

After the nine clones were purified and amplified, the prepared plasmid DNA was digested with EcoR I and Xho I and electrophoretically separated on a 0.8% agarose gel to determine if the plasmid with the inserts were successfully prepared. The alignment of the nine clones based on the restriction map and the DNA sequencing in shown in Fig. 7.

### Sequencing of the IIICS Region

The IIICS region of all nine identified clones was sequenced. There were two kinds of IIICS splicing variants present in the nine clones, one is the 120aa type (15 bp to 482 bp) in clone 185 and the other type is the 89aa type (14 bp to 282 bp) in all other eight positive clones (Additional file 3).

### Expression of ED-A fibronectin at mRNA level in SMMUpos and SMMUneg cells

**Figure 8:**
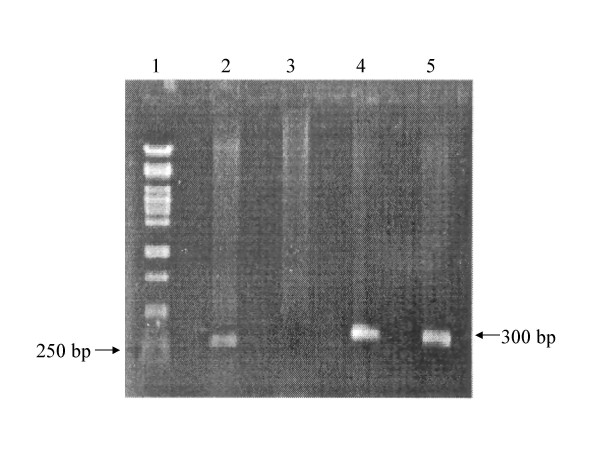
**RT-PCR of SMMUpos and SMMUneg fibronectin expression**. RT-PCR of SMMUpos and SMMUneg fibronectin expression using fibronectin primers. Lane 1: PST I DNA marker: Lane 2: Fibronectin expression in SMMUpos; Lane 3: Fibronectin expression in SMMUneg; Lane 4: β-actin expression in SMMUpos; Lane 5: β-actin expression in SMMUneg.

Fibronectins are present in all body fluids and on the cell surface of most cell types and intercellular matrices. In order to confirm if the fibronectin recognized by mAb SM5-1 expressed is the same as the one expressed on the cellular level, the mRNA expression levels of fibronectins in SMMUpos und SMMUneg cells were analyzed. Total mRNA of SMMUpos and SMMUneg was prepared and the primers were designed in the ED-A region (see material and methods) according to the human fibronectin sequence of the positive clones recognized by mAb SM5-1. Human β-actin mRNA was used as a control. The SMMUpos cell line expressed both human ED-A fibronectin and human β-actin mRNA, while the SMMUneg cell line expressed only the human β-actin mRNA (Fig. 8).

### Molecular weight of FN reactive with mAb SM 5-1

**Figure 9:**
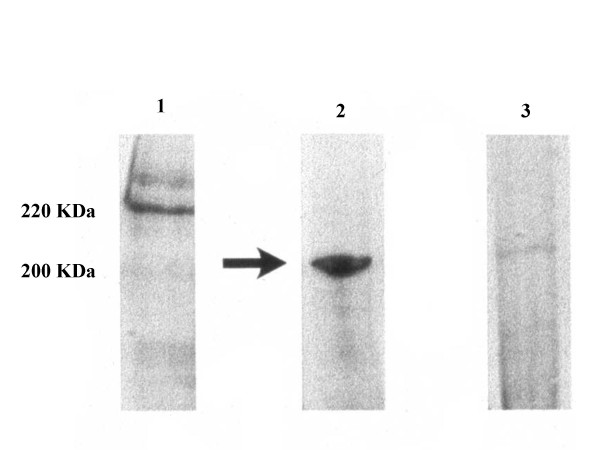
**Western blotting using mAb SM5-1**. Western blotting of the protein extracted from the SMMUpos cells with monoclonal antibody SM 5-1. 1: molecular weight standard; 2: SMMUpos; 3: SMMUneg.

In order to know the molecular weight of the fibronectin reactive with the monoclonal antibody SM 5-1, the proteins of SMMUpos and SMMUneg cells were extracted and the cell lysates run on a 5% polyacrylamide gel, blotted onto nitrocellulose membrane and immunostained with mAb SM 5-1. The molecular weight of FN expressed in SMMUpos is about 200 KDa, which is in line with the expected molecular weight [8](Fig. 9). SMMUneg cells were negative, as expected.

## Discussion

By screening a cDNA library generated from the metastatic melanoma cell line SMMUpos, the highly sensitive monoclonal antibody SM5-1 was found to recognize two fibronectin isoforms with the ED-A and CS1 regions. Fibronectins are high molecule weight adhesive glycoproteins present in soluble form in plasma (plasma fibronectin, heterodimeric), other body fluids and in insoluble form in the extracellular matrix, basal lamina as well as on cell surface (cellular fibronectin, heterodimers or multimers). Both forms have similar but not identical subunits of 200–280 KDa, which are made up of a series of repeating units of three types and joined by two disulfide bounds at their carboxyl terminus [8,9]. Each subunit of plasma and cellular FN shows considerable heterogeneity in charge and size which is accounted for by alternative splicing and variable posttranslational modifications, e.g. glycosylation, phosphorylation and sulphation [10,11]. The fibronectin variant epitope recognized by mAb SM5-1 has also to be posttranslationally modified since some of the melanoma cell lines examined do not express the standard fibronectin form, but do express a FN form recognized by SM5-1, suggesting that a melanoma-associated variant with posttranslational modification is widely expressed in melanoma. Each polypeptide subunit of FN is composed of type I, type II, and type III homology repeats containing specific sites for binding to cells and a range of molecules, including collagen, fibrils, and heparin [12-14]. Alternative splicing is seen within the central run of type III repeats. ED-A and ED-B can be included or excluded from FN mRNA by Exon skipping [8,15-18]. A third nonhomologous region called V (for variable) or IIICS (type III connecting segment) can be subdivided in humans in the 5' V25 segment and the 3' V31 segment being spliced independently of the central 64 amino acids to produce 5 potential variants [15,19]. Borsi et al. have demonstrated that FNs from transformed or tumor derived cells are composed of a population of molecules in which both the IIICS and ED-A sequences are expressed more than in FNs from normal cells [20].

The multiple functions of fibronectin include the establishment and maintenance of normal cell morphology, promotion of cell migration and enhancement of cell attachment, onto- and oncogenic transformation [21-26]. The functional role of fibronectin in relation to the biological behavior of the melanoma is open to speculation but functionally active integrin alpha(5) and fibronectin seems to be instrumental in melanoma metastases [27]. Certain short peptide sequences in fibronectin, such as RGD peptide in the type III domain, the LDV sequence in the CS1 region, the REDV peptide sequence in the CS5 region produced by the alternative splicing of the IIICS region of the fibronectin and the peptides I and II in the heparin -binding domain of the fibronectin contribute to the process of tumor cell adhesion and migration [28-34].

In the immunoscreening with mAb SM5-1 the mAb recognized two fibronectin isoforms without ED-B region in any of the nine clones, but the shortest clone, which spanned the sequence of fibronectin from 4503 bp to the 3' end, included the ED-A region. It was reported that in metastatic melanoma, only ED-A fibronectin was found, but no ED-B fibronectin [35]. This finding is consistent with our results of the RT-PCR experiment (Fig. 8). Xia et al. showed that the ED-A region increases the adhesive properties of fibronectin [36]. Therefore, one likely explanation for the in vivo effects of ED-A fibronectin on metastasis is that cells expressing high levels of ED-A fibronectin in the primary tumor will display a greater degree of homotypic adhesion in the primary melanoma and consequently fewer melanoma cells will exit the primary site and circulate to other tissues. Full length recombinant FNs including ED-A are incorporated into pericellular matrices more effectively than forms lacking ED-A [37]. Early work on fibronectin showed that fibronectin is deposited into the extracellular matrix of normal cells but that malignant counterparts of such cells often failed to deposit a fibronectin matrix [38,39].

The make-up of the different FN isoforms depends on the FN source and the alternative splicing of the FN pre-mRNA is regulated in a cell-, tissue-, and development-specific manner [17,40-45]. Alternative splicing may represent an important flexible mechanism and be able to generate diversity in a reversible fashion in response to developmental and environmental cues without requiring the expression of new genes. The FN containing ED-A segment is expressed 10 times higher in the tissue culture medium of tumor derived or SV-40 transformed human cells than that from normal human fibroblasts [20]. It was suggested that for some cell types regulation of the adhesion-promoting activity of FN may occur by alternative RNA splicing in the IIICS region [46]. The sequence of the minimal cell recognition site in the "cell-binding" domain of fibronectin has been identified as the tetrapeptide Arg-Gly-Asp-Ser (RGDS) [47,48]. The peptide RGDS and related short RGD-containing peptides have been found to inhibit both cell adhesion to fibronectin in vitro and experimental metastasis in vivo, also in a melanoma model [46,49,50]. The RGD motif was not included in our sequence but there are two other attachment sites, one is the REDV, which is somehow related to RGDS, present in the IIICS region and designed by Humphries's group as CS5 peptide and the another one is the EILDVPST peptide or CS1. It was shown that the CS1 peptide of fibronectin, lacking the RGD motif, actively inhibited tumor metastasis in spontaneous and experimental metastasis models [50].

The IIICS region is one of the alternative splicing regions that generate multiple fibronectin mRNAs. This region corresponds to the non-homologous segment which connects the last two type III units at the C-terminal end of the protein (IIICS). Fibronectin cDNAs differ by the presence or absence of IIICS segments of 285 bps or 360 bps, encoding 95 or 120 amino acids, respectively [18]. There are however five types of alternative splicing in the IIICS region, two of them (120aa type and 89aa type) include the CS1 peptide and both of which were found. Clone 185 has the 120aa type of the alternative splicing of the IIICS and the other eight clones have the 89aa type of the IIICS alternative splicing. Competitive inhibition assays and avidity determinations suggested that the CS1 region may be the major site of interaction with the melanoma cell surface [46,51].

In malignant cells, the matrix deposition is disturbed and fibronectin is absent from the matrix of many tumorigenic cell lines [47]. Three events appear to be important: the binding of fibronectin to cell surface receptors, the binding between individual fibronectin molecules and cross-linking between fibronectin molecules [52,53]. The malignant cells are incapable of depositing a fibronectin matrix but still adhere almost normally to fibronectin. Moreover, they produce fibronectin that is unaltered in the sense that normal cells can incorporate it into their matrix [26]. To these paradoxical observations there is still no sound explanation. It is also reported that the addition of exogenous fibronectin restores the fibronectin matrix and cytoskeletal organization in cultures of cells that are incapable of assembling a matrix when cultured in a normal medium [53,54]. For metastatic melanoma cells, presence of ED-A and CS1 may give them the ability to be quickly arrested in the vasculature at secondary sites and pass through the surrounding tissue. The adhesive molecules act independently or in concert to direct melanoma cells to particular tissue and grow at the secondary site. The metastases are then established.

## Conclusion

The mAb SM5-1 does not recognize a metastases-associated antigen as originally thought but react with a significantly higher percentage of melanoma specimens than any other melanoma associated antibody. The epitope recognized by mAb SM5-1 is a melanoma-associated fibronectin variant. More detailed analyses of possible post-translational modifications and functional studies are indicated on this epitope which is possibly involved in metastatic spread. ED-B FN can be used as a target for immunotherapy and antibodies to ED-B can be used both diagnostically or therapeutically in various malignancies [55,56]. It seems therefore to be feasible to potentially use the ED-A variant recognized by mAb SM5-1 for the same purposes.

## Competing interests

The author(s) declare that they have no competing interests.

## Authors' contributions

UT and WS designed the study and were instrumental in data assessment. YG generated the antibody, YC, GH and UT conducted the study. FK performed the FACS analyses. UT and YC, wrote the manuscript, WS and MAH critically discussed the findings and impacted in writing and final discussion of the manuscript.

## Pre-publication history

The pre-publication history for this paper can be accessed here:



## Supplementary Material

Additional File 1 **Primer sequences**. Sequences of the primers used for the sequence reactions. Click here for file

Additional File 2 **Sequence of the nine clones**. Complete nucleotide and deduced amino acid sequence of human fibronectin precursor based on the human pre-mRNA for fibronectin from Genbank (blast-program, accession number: X02761). The primers used to extend the nucleotide sequence of the positive clones are marked in bold and italic. The underlined sequence is the extra-domain A region and RGD motif. The underlined single bps are arrowheaded and represent the start of each clone. Click here for file

Additional File 3 **IIICS region of fibronectin**. Human fibronectin gene IIICS region for extra domain (IIICS = type III connecting strand). Genbank Accession NR. X04530. Arrowheads are intron-exon junctions. The 93 bp intron which can be part of an exon and whose presences in our positive clones were different is underlined. a: alternative splicing acceptor site; d: alternative splicing donor site. 1......14 bp: intron. 14......15: intron-exon junction, a_1_: acceptor site. 15......481: complex fibronectin IIICS exon and coding region containing different splice sites. 89......90: intron-exon junction, a_2 _acceptor site. 282......283: exon-intron junction, d_1 _donor site. 282......374: intron sequence or alternatively part of exon by alternate splicing. 374: intron-exon junction, a_3 _acceptor site. 481......482: exon-intron junction, d_2 _donor site. 482......1052: intron. In our positive clones, clone 185 containing the alternative part of exon (282......374) and other eight positive clones have the exon from the 15 bp to 481 bp excluding the exon from 282 bp to 374 bp. Click here for file

## References

[B1] Guo Y, Ma J, Wang J, Che X, Narula J, Bigby M, Wu M, Sy MS (1994). Inhibition of human melanoma growth and metastasis in vivo by anti-CD44 monoclonal antibody. Cancer Res.

[B2] Trefzer U, Rietz N, Chen Y, Audring H, Herberth G, Siegel P, Reinke S, Koniger P, Wu S, Ma J, Liu Y, Wang H, Sterry W, Guo Y (2000). SM5-1: a new monoclonal antibody which is highly sensitive and specific for melanocytic lesions. Arch Dermatol Res.

[B3] Reinke S, Koeniger P, Herberth G, Audring H, Wang H, Ma J, Guo Y, Sterry W, Trefzer U (2005). Differential expression of MART-1, Tyrosinase and SM5-1 in primary and metastatic melanoma. Am J Dermatopathol.

[B4] Dear TN, McDonald DA, Kefford RF (1989). Transcriptional down-regulation of a rat gene, WDNM2, in metastatic DMBA-8 cells. Cancer Res.

[B5] Kalebic T, Williams JE, Talmadge JE, Kao-Shan CS, Kravitz B, Locklear K, Siegal GP, Liotta LA, Sobel ME, Steeg PS (1988). A novel method for selection of invasive tumor cells: derivation and characterization of highly metastatic K1735 melanoma cell lines based on in vitro and in vivo invasive capacity. Clin Exp Metastasis.

[B6] Jacob AN, Kalapurakal J, Davidson WR, Kandpal G, Dunson N, Prashar Y, Kandpal RP (1999). Tyrosine kinase, UFO/Axl, and other genes isolated by a modified differential display PCR are overexpressed in metastatic prostatic carcinoma cell line DU145. Cancer Detect Prev.

[B7] Short JM, Fernandez JM, Sorge JA, Huse WD (1988). Lambda ZAP: a bacteriophage lambda expression vector with in vivo excision properties. Nucleic Acids Res.

[B8] Hynes RO (1985). Fibronectins: a family of complex and versatile adhesive glycoproteins derived from a single gene. Harvey Lect.

[B9] Pankov R, Cukierman E, Katz BZ, Matsumoto K, Lin DC, Lin S, Hahn C, Yamada KM (2000). Integrin dynamics and matrix assembly: tensin-dependent translocation of alpha(5)beta(1) integrins promotes early fibronectin fibrillogenesis. J Cell Biol.

[B10] Johnson KJ, Sage H, Briscoe G, Erickson HP (1999). The compact conformation of fibronectin is determined by intramolecular ionic interactions. J Biol Chem.

[B11] Paul JI, Schwarzbauer JE, Tamkun JW, Hynes RO (1986). Cell-type-specific fibronectin subunits generated by alternative splicing. J Biol Chem.

[B12] Engel J, Odermatt E, Engel A, Madri JA, Furthmayr H, Rohde H, Timpl R (1981). Shapes, domain organizations and flexibility of laminin and fibronectin, two multifunctional proteins of the extracellular matrix. J Mol Biol.

[B13] Garcia-Pardo A, Rostagno A, Frangione B (1987). Primary structure of human plasma fibronectin. Characterization of a 38 kDa domain containing the C-terminal heparin-binding site (Hep III site) and a region of molecular heterogeneity. Biochem J.

[B14] Patel RS, Odermatt E, Schwarzbauer JE, Hynes RO (1987). Organization of the fibronectin gene provides evidence for exon shuffling during evolution. EMBO J.

[B15] Kornblihtt AR, Vibe-Pedersen K, Baralle FE (1984). Human fibronectin: molecular cloning evidence for two mRNA species differing by an internal segment coding for a structural domain. EMBO J.

[B16] Kornblihtt AR, Umezawa K, Vibe-Pedersen K, Baralle FE (1985). Primary structure of human fibronectin: differential splicing may generate at least 10 polypeptides from a single gene. EMBO J.

[B17] Norton PA, Hynes RO (1987). Alternative splicing of chicken fibronectin in embryos and in normal and transformed cells. Mol Cell Biol.

[B18] Schwarzbauer JE, Patel RS, Fonda D, Hynes RO (1987). Multiple sites of alternative splicing of the rat fibronectin gene transcript. EMBO J.

[B19] Sekiguchi K, Klos AM, Hirohashi S, Hakomori S (1986). Human tissue fibronectin: expression of different isotypes in the adult and fetal tissues. Biochem Biophys Res Commun.

[B20] Borsi L, Carnemolla B, Castellani P, Rosellini C, Vecchio D, Allemanni G, Chang SE, Taylor-Papadimitriou J, Pande H, Zardi L (1987). Monoclonal antibodies in the analysis of fibronectin isoforms generated by alternative splicing of mRNA precursors in normal and transformed human cells. J Cell Biol.

[B21] Wierzbicka-Patynowski I, Schwarzbauer JE (2003). The ins and outs of fibronectin matrix assembly. J Cell Sci.

[B22] Ugarova TP, Ljubimov AV, Deng L, Plow EF (1996). Proteolysis regulates exposure of the IIICS-1 adhesive sequence in plasma fibronectin. Biochemistry.

[B23] Sechler JL, Cumiskey AM, Gazzola DM, Schwarzbauer JE (2000). A novel RGD-independent fibronectin assembly pathway initiated by alpha4beta1 integrin binding to the alternatively spliced V region. J Cell Sci.

[B24] Ohashi T, Kiehart DP, Erickson HP (1999). Dynamics and elasticity of the fibronectin matrix in living cell culture visualized by fibronectin-green fluorescent protein. Proc Natl Acad Sci U S A.

[B25] Hormann H (1982). Fibronectin – mediator between cells and connective tissue. Klin Wochenschr.

[B26] Ruoslahti E, Pierschbacher MD (1987). New perspectives in cell adhesion: RGD and integrins. Science.

[B27] Qian F, Zhang ZC, Wu XF, Li YP, Xu Q (2005). Interaction between integrin alpha(5) and fibronectin is required for metastasis of B16F10 melanoma cells. Biochem Biophys Res Commun.

[B28] Cukierman E, Pankov R, Stevens DR, Yamada KM (2001). Taking cell-matrix adhesions to the third dimension. Science.

[B29] Manabe R, Oh-e N, Sekiguchi K (1999). Alternatively spliced EDA segment regulates fibronectin-dependent cell cycle progression and mitogenic signal transduction. J Biol Chem.

[B30] Ingham KC, Brew SA, Huff S, Litvinovich SV (1997). Cryptic self-association sites in type III modules of fibronectin. J Biol Chem.

[B31] Morla A, Zhang Z, Ruoslahti E (1994). Superfibronectin is a functionally distinct form of fibronectin. Nature.

[B32] Sechler JL, Schwarzbauer JE (1997). Coordinated regulation of fibronectin fibril assembly and actin stress fiber formation. Cell Adhes Commun.

[B33] Sechler JL, Takada Y, Schwarzbauer JE (1996). Altered rate of fibronectin matrix assembly by deletion of the first type III repeats. J Cell Biol.

[B34] Ugarova TP, Zamarron C, Veklich Y, Bowditch RD, Ginsberg MH, Weisel JW, Plow EF (1995). Conformational transitions in the cell binding domain of fibronectin. Biochemistry.

[B35] Natali PG, Nicotra MR, Di Filippo F, Bigotti A (1995). Expression of fibronectin, fibronectin isoforms and integrin receptors in melanocytic lesions. Br J Cancer.

[B36] Xia P, Culp LA (1995). Adhesion activity in fibronectin's alternatively spliced domain EDa (EIIIA): complementarity to plasma fibronectin functions. Exp Cell Res.

[B37] Guan JL, Trevithick JE, Hynes RO (1990). Retroviral expression of alternatively spliced forms of rat fibronectin. J Cell Biol.

[B38] Hynes RO, George EL, Georges EN, Guan JL, Rayburn H, Yang JT (1992). Toward a genetic analysis of cell-matrix adhesion. Cold Spring Harb Symp Quant Biol.

[B39] Ruoslahti E (1984). Fibronectin in cell adhesion and invasion. Cancer Metastasis Rev.

[B40] Barone MV, Henchcliffe C, Baralle FE, Paolella G (1989). Cell type specific trans-acting factors are involved in alternative splicing of human fibronectin pre-mRNA. EMBO J.

[B41] Carnemolla B, Balza E, Siri A, Zardi L, Nicotra MR, Bigotti A, Natali PG (1989). A tumor-associated fibronectin isoform generated by alternative splicing of messenger RNA precursors. J Cell Biol.

[B42] Ffrench-Constant C, Hynes RO (1989). Alternative splicing of fibronectin is temporally and spatially regulated in the chicken embryo. Development.

[B43] Glukhova MA, Frid MG, Shekhonin BV, Balabanov YV, Koteliansky VE (1990). Expression of fibronectin variants in vascular and visceral smooth muscle cells in development. Dev Biol.

[B44] Laitinen L, Vartio T, Virtanen I (1991). Cellular fibronectins are differentially expressed in human fetal and adult kidney. Lab Invest.

[B45] Pagani F, Zagato L, Vergani C, Casari G, Sidoli A, Baralle FE (1991). Tissue-specific splicing pattern of fibronectin messenger RNA precursor during development and aging in rat. J Cell Biol.

[B46] Humphries MJ, Olden K, Yamada KM (1986). A synthetic peptide from fibronectin inhibits experimental metastasis of murine melanoma cells. Science.

[B47] Pierschbacher MD, Ruoslahti E (1984). Cell attachment activity of fibronectin can be duplicated by small synthetic fragments of the molecule. Nature.

[B48] Pierschbacher MD, Dedhar S, Ruoslahti E, Argraves S, Suzuki S (1988). An adhesion variant of the MG-63 osteosarcoma cell line displays an osteoblast-like phenotype. Ciba Found Symp.

[B49] Humphries MJ, Yasuda Y, Olden K, Yamada KM (1988). The cell interaction sites of fibronectin in tumour metastasis. Ciba Found Symp.

[B50] Saiki I, Murata J, Iida J, Sakurai T, Nishi N, Matsuno K, Azuma I (1989). Antimetastatic effects of synthetic polypeptides containing repeated structures of the cell adhesive Arg-Gly-Asp (RGD) and Tyr-Ile-Gly-Ser-Arg (YIGSR) sequences. Br J Cancer.

[B51] Humphries MJ, Komoriya A, Akiyama SK, Olden K, Yamada KM (1987). Identification of two distinct regions of the type III connecting segment of human plasma fibronectin that promote cell type-specific adhesion. J Biol Chem.

[B52] McKeown-Longo PJ, Mosher DF (1983). Binding of plasma fibronectin to cell layers of human skin fibroblasts. J Cell Biol.

[B53] Roman J, LaChance RM, Broekelmann TJ, Kennedy CJ, Wayner EA, Carter WG, McDonald JA (1989). The fibronectin receptor is organized by extracellular matrix fibronectin: implications for oncogenic transformation and for cell recognition of fibronectin matrices. J Cell Biol.

[B54] Hayman EG, Engvall E, Ruoslahti E (1981). Concomitant loss of cell surface fibronectin and laminin from transformed rat kidney cells. J Cell Biol.

[B55] Ebbinghaus C, Scheuermann J, Neri D, Elia G (2004). Diagnostic and therapeutic applications of recombinant antibodies: targeting the extra-domain B of fibronectin, a marker of tumor angiogenesis. Curr Pharm Des.

[B56] Menrad A, Menssen HD (2005). ED-B fibronectin as a target for antibody-based cancer treatments. Expert Opin Ther Targets.

