# Supplementary material for: The monoclonal antibody SM5-1 recognizes a fibronectin variant which is widely expressed in melanoma
Source: BMC Cancer. 2006 Jan 11;6:8. doi: 10.1186/1471-2407-6-8 (PMC1351261; doi:10.1186/1471-2407-6-8)
Supplement: Additional File 1 — Primer sequences. Sequences of the primers used for the sequence reactions. [file 1471-2407-6-8-S1.ppt]

## Slide 1
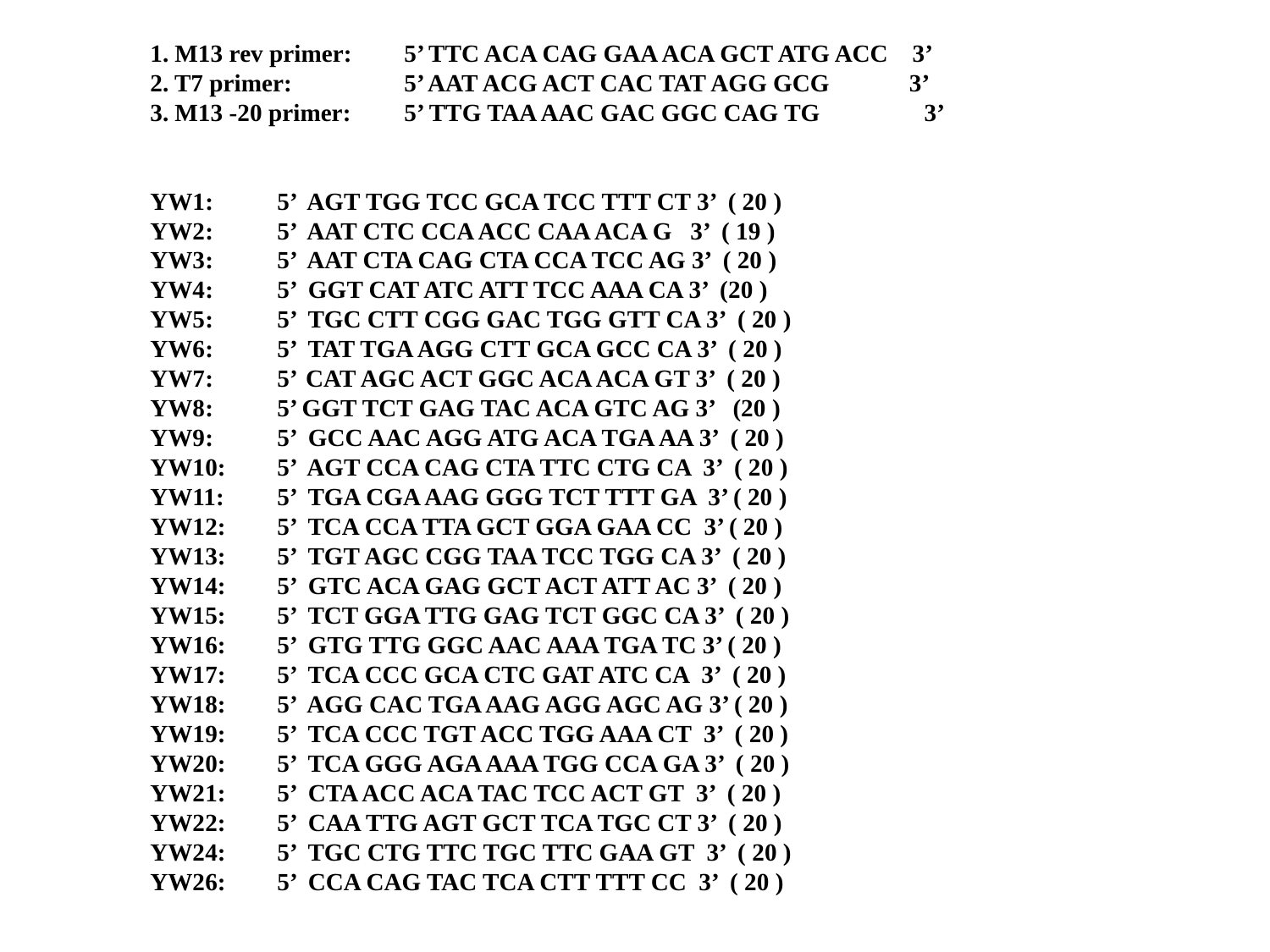

1. M13 rev primer:	5’ TTC ACA CAG GAA ACA GCT ATG ACC 3’
2. T7 primer: 	5’ AAT ACG ACT CAC TAT AGG GCG 3’
3. M13 -20 primer: 	5’ TTG TAA AAC GAC GGC CAG TG 	 3’
YW1: 	5’ AGT TGG TCC GCA TCC TTT CT 3’ ( 20 )
YW2: 	5’ AAT CTC CCA ACC CAA ACA G 3’ ( 19 )
YW3: 	5’ AAT CTA CAG CTA CCA TCC AG 3’ ( 20 )
YW4: 	5’ GGT CAT ATC ATT TCC AAA CA 3’ (20 )
YW5: 	5’ TGC CTT CGG GAC TGG GTT CA 3’ ( 20 )
YW6: 	5’ TAT TGA AGG CTT GCA GCC CA 3’ ( 20 )
YW7: 	5’ CAT AGC ACT GGC ACA ACA GT 3’ ( 20 )
YW8: 	5’ GGT TCT GAG TAC ACA GTC AG 3’ (20 )
YW9: 	5’ GCC AAC AGG ATG ACA TGA AA 3’ ( 20 )
YW10: 	5’ AGT CCA CAG CTA TTC CTG CA 3’ ( 20 )
YW11: 	5’ TGA CGA AAG GGG TCT TTT GA 3’ ( 20 )
YW12: 	5’ TCA CCA TTA GCT GGA GAA CC 3’ ( 20 )
YW13: 	5’ TGT AGC CGG TAA TCC TGG CA 3’ ( 20 )
YW14: 	5’ GTC ACA GAG GCT ACT ATT AC 3’ ( 20 )
YW15: 	5’ TCT GGA TTG GAG TCT GGC CA 3’ ( 20 )
YW16: 	5’ GTG TTG GGC AAC AAA TGA TC 3’ ( 20 )
YW17: 	5’ TCA CCC GCA CTC GAT ATC CA 3’ ( 20 )
YW18: 	5’ AGG CAC TGA AAG AGG AGC AG 3’ ( 20 )
YW19: 	5’ TCA CCC TGT ACC TGG AAA CT 3’ ( 20 )
YW20: 	5’ TCA GGG AGA AAA TGG CCA GA 3’ ( 20 )
YW21: 	5’ CTA ACC ACA TAC TCC ACT GT 3’ ( 20 )
YW22: 	5’ CAA TTG AGT GCT TCA TGC CT 3’ ( 20 )
YW24: 	5’ TGC CTG TTC TGC TTC GAA GT 3’ ( 20 )
YW26: 	5’ CCA CAG TAC TCA CTT TTT CC 3’ ( 20 )
