# Supplementary material for: The monoclonal antibody SM5-1 recognizes a fibronectin variant which is widely expressed in melanoma
Source: BMC Cancer. 2006 Jan 11;6:8. doi: 10.1186/1471-2407-6-8 (PMC1351261; doi:10.1186/1471-2407-6-8)
Supplement: Additional File 2 — Sequence of the nine clones. Complete nucleotide and deduced amino acid sequence of human fibronectin precursor based on the human pre-mRNA for fibronectin from Genbank (blast-program, accession number: X02761). The primers used to extend the nucleotide sequence of the positive clones are marked in bold and italic. The underlined sequence is the extra-domain A region and RGD motif. The underlined single bps are arrowheaded and represent the start of each clone. [file 1471-2407-6-8-S2.doc]

clone 146(1998)

CTCGCTTTGACTTCACCACCACCAGCACCAGCACACCTGTGAC

--+---------+---------+---------+---------+ 2040

GAGCGAAACTGAAGTGGTGGTGGTCGTGGTCGTGTGGACACTG

T R F D F T T T S T S T P V T -

CAGCAACACCGTGACAGGAGAGACGACTCCCTTTTCTCCTCTTGTGGCCACTTCTGAATC

2041 ---------+---------+---------+---------+---------+---------+ 2100

GTCGTTGTGGCACTGTCCTCTCTGCTGAGGGAAAAGAGGAGAACACCGGTGAAGACTTAG

S N T V T G E T T P F S P L V A T S E S -

TGTGACCGAAATCACAGCCAGTAGCTTTGTGGTCTCCTGGGTCTCAGCTTCCGACACCGT

2101 ---------+---------+---------+---------+---------+---------+ 2160

ACACTGGCTTTAGTGTCGGTCATCGAAACACCAGAGGACCCAGAGTCGAAGGCTGTGGCA

V T E I T A S S F V V S W V S A S D T V -

clone 185-1(2190)

GTCGGGATTCCGGGTGGAATATGAGCTGAGTGAGGAGGGAGATGAGCCACAGTACCTGGA

2161 ---------+---------+---------+---------+---------+---------+ 2220

CAGCCCTAAGGCCCACCTTATACTCGACTCACTCCTCCCTCTACTCGGTGTCATGGACCT

S G F R V E Y E L S E E G D E P Q Y L D -

TCTTCCAAGCACAGCCACTTCTGTGAACATCCCTGACCTGCTTCCTGGCCGAAAATACAT

2221 ---------+---------+---------+---------+---------+---------+ 2280

AGAAGGTTCGTGTCGGTGAAGACACTTGTAGGGACTGGACGAAGGACCGGCTTTTATGTA

L P S T A T S V N I P D L L P G R K Y I -

clone 127-1a (2319)

TGTAAATGTCTATCAGATATCTGAGGATGGGGAGCAGAGTTTGATCCTGTCTACTTCACA

2281 ---------+---------+---------+---------+---------+---------+ 2340

ACATTTACAGATAGTCTATAGACTCCTACCCCTCGTCTCAAACTAGGACAGATGAAGTGT

V N V Y Q I S E D G E Q S L I L S T S Q -

AACAACAGCGCCTGATGCCCCTCCTGACCCGACTGTGGACCAAGTTGATGACACCTCAAT

2341 ---------+---------+---------+---------+---------+---------+ 2400

TTGTTGTCGCGGACTACGGGGAGGACTGGGCTGACACCTGGTTCAACTACTGTGGAGTTA

T T A P D A P P D P T V D Q V D D T S I -

TGTTGTTCGCTGGAGCAGACCCCAGGCTCCCATCACAGGGTACAGAATAGTCTATTCGCC

2401 ---------+---------+---------+---------+---------+---------+ 2460

ACAACAAGCGACCTCGTCTGGGGTCCGAGGGTAGTGTCCCATGTCTTATCAGATAAGCGG

V V R W S R P Q A P I T G Y R I V Y S P -

ATCAGTAGAAGGTAGCAGCACAGAACTCAACCTTCCTGAAACTGCAAACTCCGTCACCCT

2461 ---------+---------+---------+---------+---------+---------+ 2520

TAGTCATCTTCCATCGTCGTGTCTTGAGTTGGAAGGACTTTGACGTTTGAGGCAGTGGGA

S V E G S S T E L N L P E T A N S V T L -

CAGTGACTTGCAACCTGGTGTTCAGTATAACATCACTATCTATGCTGTGGAAGAAAATCA

2521 ---------+---------+---------+---------+---------+---------+ 2580

GTCACTGAACGTTGGACCACAAGTCATATTGTAGTGATAGATACGACACCTTCTTTTAGT

S D L Q P G V Q Y N I T I Y A V E E N Q -

AGAAAGTACACCTGTTGTCATTCAACAAGAAACCACTGGCACCCCACGCTCAGATACAGT

2581 ---------+---------+---------+---------+---------+---------+ 2640

TCTTTCATGTGGACAACAGTAAGTTGTTCTTTGGTGACCGTGGGGTGCGAGTCTATGTCA

E S T P V V I Q Q E T T G T P R S D T V -

clone 130-1 and clone 181-2 (2652)

GCCCTCTCCCAGGGACCTGCAGTTTGTGGAAGTGACAGACGTGAAGGTCACCATCATGTG

2641 ---------+---------+---------+---------+---------+---------+ 2700

CGGGAGAGGGTCCCTGGACGTCAAACACCTTCACTGTCTGCACTTCCAGTGGTAGTACAC

P S P R D L Q F V E V T D V K V T I M W -

GACACCGCCTGAGAGTGCAGTGACCGGCTACCGTGTGGATGTGATCCCCGTCAACCTGCC

2701 ---------+---------+---------+---------+---------+---------+ 2760

CTGTGGCGGACTCTCACGTCACTGGCCGATGGCACACCTACACTAGGGGCAGTTGGACGG

T P P E S A V T G Y R V D V I P V N L P -

TGGCGAGCACGGGCAGAGGCTGCCCATCAGCAGGAACACCTTTGCAGAAGTCACCGGGCT

2761 ---------+---------+---------+---------+---------+---------+ 2820

ACCGCTCGTGCCCGTCTCCGACGGGTAGTCGTCCTTGTGGAAACGTCTTCAGTGGCCCGA

G E H G Q R L P I S R N T F A E V T G L -

clone 187-1 (2838)

GTCCCCTGGGGTCACC**T**ATTACTTCAAAGTCTTTGCAGTGAGCCATGGGAGGGAGAGCAA

2821 ---------+---------+---------+---------+---------+---------+ 2880

CAGGGGACCCCAGTGGATAATGAAGTTTCAGAAACGTCACTCGGTACCCTCCCTCTCGTT

S P G V T Y Y F K V F A V S H G R E S K -

GCCTCTGACTGCTCAACAGACAACCAAACTGGATGCTCCCACTAACCTCCAGTTTGTCAA

2881 ---------+---------+---------+---------+---------+---------+ 2940

CGGAGACTGACGAGTTGTCTGTTGGTTTGACCTACGAGGGTGATTGGAGGTCAAACAGTT

P L T A Q Q T T K L D A P T N L Q F V N -

TGAAACTGATTCTACTGTCCTGGTGAGATGGACTCCACCTCGGGCCCAGATAACAGGATA

2941 ---------+---------+---------+---------+---------+---------+ 3000

ACTTTGACTAAGATGACAGGACCACTCTACCTGAGGTGGAGCCCGGGTCTATTGTCCTAT

E T D S T V L V R W T P P R A Q I T G Y -

CCGACTGACCGTGGGCCTTACCCGAAGAGGCCAGCCCAGGCAGTACAATGTGGGTCCCTC

3001 ---------+---------+---------+---------+---------+---------+ 3060

GGCTGACTGGCACCCGGAATGGGCTTCTCCGGTCGGGTCCGTCATGTTACACCCAGGGAG

R L T V G L T R R G Q P R Q Y N V G P S -

TGTCTCCAAGTACCCCCTGAGGAATCTGCAGCCTGCATCTGAGTACACCGTATCCCTCGT

3061 ---------+---------+---------+---------+---------+---------+ 3120

ACAGAGGTTCATGGGGGACTCCTTAGACGTCGGACGTAGACTCATGTGGCATAGGGAGCA

V S K Y P L R N L Q P A S E Y T V S L V -

GGCCATAAAGGGCAACCAAGAGAGCCCCAAAGCCACTGGAGTCTTTACCACACTGCAGCC

3121 ---------+---------+---------+---------+---------+---------+ 3180

CCGGTATTTCCCGTTGGTTCTCTCGGGGTTTCGGTGACCTCAGAAATGGTGTGACGTCGG

A I K G N Q E S P K A T G V F T T L Q P -

TGGGAGCTCTATTCCACCTTACAACACCGAGGTGACTGAGACCACCATCGTGATCACATG

3181 ---------+---------+---------+---------+---------+---------+ 3240

ACCCTCGAGATAAGGTGGAATGTTGTGGCTCCACTGACTCTGGTGGTAGCACTAGTGTAC

G S S I P P Y N T E V T E T T I V I T W -

GACGCCTGCTCCAAGAATTGGTTTTAAGCTGGGTGTACGACCAAGCCAGGGAGGAGAGGC

3241 ---------+---------+---------+---------+---------+---------+ 3300

CTGCGGACGAGGTTCTTAACCAAAATTCGACCCACATGCTGGTTCGGTCCCTCCTCTCCG

T P A P R I G F K L G V R P S Q G G E A -

ACCACGAGAAGTGACTTCAGACTCAGGAAGCATCGTTGTGTCCGGCTTGACTCCAGGAGT

3301 ---------+---------+---------+---------+---------+---------+ 3360

TGGTGCTCTTCACTGAAGTCTGAGTCCTTCGTAGCAACACAGGCCGAACTGAGGTCCTCA

P R E V T S D S G S I V V S G L T P G V -

AGAATACGTCTACACCATCCAAGTCCTGAGAGATGGACAGGAAAGAGATGCGCCAATTGT

3361 ---------+---------+---------+---------+---------+---------+ 3420

TCTTATGCAGATGTGGTAGGTTCAGGACTCTCTACCTGTCCTTTCTCTACGCGGTTAACA

E Y V Y T I Q V L R D G Q E R D A P I V -

AAACAAAGTGGTGACACCATTGTCTCCACCAACAAACTTGCATCTGGAGGCAAACCCTGA

3421 ---------+---------+---------+---------+---------+---------+ 3480

TTTGTTTCACCACTGTGGTAACAGAGGTGGTTGTTTGAACGTAGACCTCCGTTTGGGACT

N K V V T P L S P P T N L H L E A N P D -

CACTGGAGTGCTCACAGTCTCCTGGGAGAGGAGCACCACCCCAGACATTACTGGTTATAG

3481 ---------+---------+---------+---------+---------+---------+ 3540

GTGACCTCACGAGTGTCAGAGGACCCTCTCCTCGTGGTGGGGTCTGTAATGACCAATATC

T G V L T V S W E R S T T P D I T G Y R -

AATTACCACAACCCCTACAAACGGCCAGCAGGGAAATTCTTTGGAAGAAGTGGTCCATGC

3541 ---------+---------+---------+---------+---------+---------+ 3600

TTAATGGTGTTGGGGATGTTTGCCGGTCGTCCCTTTAAGAAACCTTCTTCACCAGGTACG

I T T T P T N G Q Q G N S L E E V V H A -

TGATCAGAGCTCCTGCACTTTTGATAACCTGAGTCCCGGCCTGGAGTACAATGTCAGTGT

3601 ---------+---------+---------+---------+---------+---------+ 3660

ACTAGTCTCGAGGACGTGAAAACTATTGGACTCAGGGCCGGACCTCATGTTACAGTCACA

D Q S S C T F D N L S P G L E Y N V S V -

TTACACTGTCAAGGATGACAAGGAAAGTGTCCCTATCTCTGATACCATCATCCCAGCTGT

3661 ---------+---------+---------+---------+---------+---------+ 3720

AATGTGACAGTTCCTACTGTTCCTTTCACAGGGATAGAGACTATGGTAGTAGGGTCGACA

Y T V K D D K E S V P I S D T I I P A V -

TCCTCCTCCCACTGACCTGCGATTCACCAACATTGGTCCAGACACCATGCGTGTCACCTG

3721 ---------+---------+---------+---------+---------+---------+ 3780

AGGAGGAGGGTGACTGGACGCTAAGTGGTTGTAACCAGGTCTGTGGTACGCACAGTGGAC

P P P T D L R F T N I G P D T M R V T W -

clone 157-1-1 (3828)

GGCTCCACCCCCATCCATTGATTTAACCAACTTCCTGGTGCGTTACTCACCTGTGAAAAA

3781 ---------+---------+---------+---------+---------+---------+ 3840

CCGAGGTGGGGGTAGGTAACTAAATTGGTTGAAGGACCACGCAATGAGTGGACACTTTTT

A P P P S I D L T N F L V R Y S P V K N -

TGAGGAAGATGTTGCAGAGTTGTCAATTTCTCCTTCAGACAATGCAGTGGTCTTAACAAA

3841 ---------+---------+---------+---------+---------+---------+ 3900

ACTCCTTCTACAACGTCTCAACAGTTAAAGAGGAAGTCTGTTACGTCACCAGAATTGTTT

E E D V A E L S I S P S D N A V V L T N -

clone 131-1-1 (3952)

TCTCCTGCCTGGTACAGAATATGTAGTGAGTGTCTCCAGTGTCTACGAACAACATGAGAG

3901 ---------+---------+---------+---------+---------+---------+ 3960

AGAGGACGGACCATGTCTTATACATCACTCACAGAGGTCACAGATGCTTGTTGTACTCTC

L L P G T E Y V V S V S S V Y E Q H E S -

CACACCTCTTAGAGGAAGACAGAAAACAGGTCTTGATTCCCCAACTGGCATTGACTTTTC

3961 ---------+---------+---------+---------+---------+---------+ 4020

GTGTGGAGAATCTCCTTCTGTCTTTTGTCCAGAACTAAGGGGTTGACCGTAACTGAAAAG

T P L R G R Q K T G L D S P T G I D F S -

TGATATTACTGCCAACTCTTTTACTGTGCACTGGATTGCTCCTCGAGCCACCATCACTGG

4021 ---------+---------+---------+---------+---------+---------+ 4080

ACTATAATGACGGTTGAGAAAATGACACGTGACCTAACGAGGAGCTCGGTGGTAGTGACC

D I T A N S F T V H W I A P R A T I T G -

CTACAGGATCCGCCATCATCCCGAGCACTTCAGTGGGAGACCTCGAGAAGATCGGGTGCC

4081 ---------+---------+---------+---------+---------+---------+ 4140

GATGTCCTAGGCGGTAGTAGGGCTCGTGAAGTCACCCTCTGGAGCTCTTCTAGCCCACGG

Y R I R H H P E H F S G R P R E D R V P -

clone 139-1 (4179)

CCACTCTCGGAATTCCATCACCCTCACCAACCTCACTCCAGGCACAGAGTATGTGGTCAG

4141 ---------+---------+---------+---------+---------+---------+ 4200

GGTGAGAGCCTTAAGGTAGTGGGAGTGGTTGGAGTGAGGTCCGTGTCTCATACACCAGTC

H S R N S I T L T N L T P G T E Y V V S -

CATCGTTGCTCTTAATGGCAGAGAGGAAAGTCCCTTATTGATTGGCCAACAATCAACAGT

4201 ---------+---------+---------+---------+---------+---------+ 4260

GTAGCAACGAGAATTACCGTCTCTCCTTTCAGGGAATAACTAACCGGTTGTTAGTTGTCA

I V A L N G R E E S P L L I G Q Q S T V -

TTCTGATGTTCCGAGGGACCTGGAAGTTGTTGCTGCGACCCCCACCAGCCTACTGATCAG

4261 ---------+---------+---------+---------+---------+---------+ 4320

AAGACTACAAGGCTCCCTGGACCTTCAACAACGACGCTGGGGGTGGTCGGATGACTAGTC

S D V P R D L E V V A A T P T S L L I S -

CTGGGATGCTCCTGCTGTCACAGTGAGATATTACAGGATCACTTACGGAGAAACAGGAGG

4321 ---------+---------+---------+---------+---------+---------+ 4380

GACCCTACGAGGACGACAGTGTCACTCTATAATGTCCTAGTGAATGCCTCTTTGTCCTCC

W D A P A V T V R Y Y R I T Y G E T G G -

5’ primer yw3 3’

AAATAGCCCTGTCCAGGAGTTCACTGTGCCTGGGAGCA***AGTCTACAGCTACCATCAGC***GG

4381 ---------+---------+---------+---------+---------+---------+ 4440

TTTATCGGGACAGGTCCTCAAGTGACACGGACCCTCGTTCAGATGTCGATGGTAGTCGCC

N S P V Q E F T V P G S K S T A T I S G -

CCTTAAACCTGGAGTTGATTATACCATCACTGTGTATGCTGTCACTGGCCGTGGAGACAG

4441 ---------+---------+---------+---------+---------+---------+ 4500

GGAATTTGGACCTCAACTAATATGGTAGTGACACATACGACAGTGACCGGCACCTCTGTC

L K P G V D Y T I T V Y A V T G **R G D S** -

clone 184-1(4503)

CCCCGCAAGCAGCAAGCCAATTTCCATTAATTACCGAACAGAAATTGACAAACCATCCCA

4501 ---------+---------+---------+---------+---------+---------+ 4560

GGGGCGTTCGTCGTTCGGTTAAAGGTAATTAATGGCTTGTCTTTAACTGTTTGGTAGGGT

P A S S K P I S I N Y R T E I D K P S Q -

GATGCAAGTGACCGATGTTCAGGACAACAGCATTAGTGTCAAGTGGCTGCCTTCAAGTTC

4561 ---------+---------+---------+---------+---------+---------+ 4620

CTACGTTCACTGGCTACAAGTCCTGTTGTCGTAATCACAGTTCACCGACGGAAGTTCAAG

M Q V T D V Q D N S I S V K W L P S S S -

CCCTGTTACTGGTTACAGAGTAACCACCACTCCCAAAAATGGACCAGGACCAACAAAAAC

4621 ---------+---------+---------+---------+---------+---------+ 4680

GGGACAATGACCAATGTCTCATTGGTGGTGAGGGTTTTTACCTGGTCCTGGTTGTTTTTG

P V T G Y R V T T T P K N G P G P T K T -

5’ primer yw6 3’

TAAAACTGCAGGTCCAGATCAAACAGAAATGAC***TATTGAAGGCTTGCAGCCCA***CAGTGGA

4681 ---------+---------+---------+---------+---------+---------+ 4740

ATTTTGACGTCCAGGTCTAGTTTGTCTTTACTGATAACTTCCGAACGTCGGG***TGTCACCT***

3’ primer

K T A G P D Q T E M T I E G L Q P T V E -

GTATGTGGTTAGTGTCTATGCTCAGAATCCAAGCGGAGAGAGTCAGCCTCTGGTTCAGAC

4741 ---------+---------+---------+---------+---------+---------+ 4800

***CATACACCAATC***ACAGATACGAGTCTTAGGTTCGCCTCTCTCAGTCGGAGACCAAGTCTG

yw21 5’

Y V V S V Y A Q N P S G E S Q P L V Q T -

TGCAGTAACCAACATTGATCGCCCTAAAGGACTGGCATTCACTGATGTGGATGTCGATTC

4801 ---------+---------+---------+---------+---------+---------+ 4860

ACGTCATTGGTTGTAACTAGCGGGATTTCCTGACCGTAAGTGACTACACCTACAGCTAAG

A V T N I D R P K G L A F T D V D V D S -

CATCAAAATTGCTTGGGAAAGCCCACAGGGGCAAGTTTCCAGGTACAGGGTGACCTACTC

4861 ---------+---------+---------+---------+---------+---------+ 4920

GTAGTTTTAACGAACCCTTTCGGGTGTCCCCGT***TCAAAGGTCCATGTCCCACT***GGATGAG

3’ primer yw19 5’

I K I A W E S P Q G Q V S R Y R V T Y S -

GAGCCCTGAGGATGGAATCCATGAGCTATTCCCTGCACCTGATGGTGAAGAAGACACTGC

4921 ---------+---------+---------+---------+---------+---------+ 4980

CTCGGGACTCCTACCTTAGGTACTCGATAAGGGACGTGGACTACCACTTCTTCTGTGACG

S P E D G I H E L F P A P D G E E D T A -

5’ primer yw8 3’

AGAGCTGCAAGGCCTCAGACCG***GGTTCTGAGTACACAGTCAG***TGTGGTTGCCTTGCACGA

4981 ---------+---------+---------+---------+---------+---------+ 5040

TCTCGACGTTCCGGAGTCTGGCCCAAGACTCATGTGTCAGTCACACCAACGGAACGTGCT

E L Q G L R P G S E Y T V S V V A L H D -

5’ primer yw10 3’

TGATATGGAGAGCCAGCCCCTGATTGGAACCC***AGTCCACAGCTATTCCTGCA***CCAACTGA

5041 ---------+---------+---------+---------+---------+---------+ 5100

ACTATACCTCTCGGTCGGGGACTAACCTTGGGTCAGGTGTCGATAAGGACGTGGTTGACT

D M E S Q P L I G T Q S T A I P A P T D -

CCTGAAGTTCACTCAGGTCACACCCACAAGCCTGAGCGCCCAGTGGACACCACCCAATGT

5101 ---------+---------+---------+---------+---------+---------+ 5160

GGACTTCAAGTGAGTCCAGTGTGGGTGTTCGGACTCGCGGGTCACCTGTGGTGGGTTACA

L K F T Q V T P T S L S A Q W T P P N V -

TCAGCTCACTGGATATCGAGTGCGGGTGACCCCCAAGGAGAAGACCGGACCAATGAAAGA

5161 ---------+---------+---------+---------+---------+---------+ 5220

AGTCGAGTG***ACCTATAGCTCACGCCCACT***GGGGGTTCCTCTTCTGGCCTGGTTACTTTCT

3’ primer yw 17 5’

Q L T G Y R V R V T P K E K T G P M K E -

AATCAACCTTGCTCCTGACAGCTCATCCGTGGTTGTATCAGGACTTATGGTGGCCACCAA

5221 ---------+---------+---------+---------+---------+---------+ 5280

TTAGTTGGAACGAGGACTGTCGAGTAGGCACCAACATAGTCCTGAATACCACCGGTGGTT

I N L A P D S S S V V V S G L M V A T K -

ATATGAAGTGAGTGTCTATGCTCTTAAGGACACTTTGACAAGCAGACCAGCTCAGGGTGT

5281 ---------+---------+---------+---------+---------+---------+ 5340

TATACTTCACTCACAGATACGAGAATTCCTGTGAAACTGTTCGTCTGGTCGAGTCCCACA

Y E V S V Y A L K D T L T S R P A Q G V -

TGTCACCACTCTGGAGAATGTCAGCCCACCAAGAAGGGCTCGTGTGACAGATGCTACTGA

5341 ---------+---------+---------+---------+---------+---------+ 5400

ACAGTGGTGAGACCTCTTACAGTCGGGTGGTTCTTCCCGAGCACACTGTCTACGATGACT

V T T L E N V S P P R R A R V T D A T E -

5’ primer yw12 3’

GACCACCA***TCACCATTAGCTGGAGAACC***AAGACTGAGACGATCACTGGCTTCCAAGTTGA

5401 ---------+---------+---------+---------+---------+---------+ 5460

CTGGTGGTAGTGGTAATCGACCTCTTGGTTCTGACTCTGCTAGTGACCGAAGGTTCAACT

T T I T I S W R T K T E T I T G F Q V D -

TGCCGTTCCAGCCAATGGCCAGACTCCAATCCAGAGAACCATCAAGCCAGATGTCAGAAG

5461 ---------+---------+---------+---------+---------+---------+ 5520

ACGGCAAGGTCGGTT***ACCGGTCTGAGGTTAGGTCT***CTTGGTAGTTCGGTCTACAGTCTTC

3’ primer yw 15 5’

A V P A N G Q T P I Q R T I K P D V R S -

CTACACCATCACAGGTTTACAACCAGGCACTGACTACAAGATCTACCTGTACACCTTGAA

5521 ---------+---------+---------+---------+---------+---------+ 5580

GATGTGGTAGTGTCCAAATGTTGGTCCGTGACTGATGTTCTAGATGGACATGTGGAACTT

Y T I T G L Q P G T D Y K I Y L Y T L N -

TGACAATGCTCGGAGCTCCCCTGTGGTCATCGACGCCTCCACTGCCATTGATGCACCATC

5581 ---------+---------+---------+---------+---------+---------+ 5640

ACTGTTACGAGCCTCGAGGGGACACCAGTAGCTGCGGAGGTGACGGTAACTACGTGGTAG

D N A R S S P V V I D A S T A I D A P S -

CAACCTGCGTTTCCTGGCCACCACACCCAATTCCTTGCTGGTATCATGGCAGCCGCCACG

5641 ---------+---------+---------+---------+---------+---------+ 5700

GTTGGACGCAAAGGACCGGTGGTGTGGGTTAAGGAACGACCATAGTACCGTCGGCGGTGC

N L R F L A T T P N S L L V S W Q P P R -

TGCCAGGATTACCGGCTACATCATCAAGTATGAGAAGCCTGGGTCTCCTCCCAGAGAAGT

5701 ---------+---------+---------+---------+---------+---------+ 5760

***ACGGTCCTAATGGCCGATGT***AGTAGTTCATACTCTTCGGACCCAGAGGAGGGTCTCTTCA

3’ primer yw 13 5’

A R I T G Y I I K Y E K P G S P P R E V -

5’ primer yw 14 3’

GGTCCCTCGGCCCCGCCCTGGT***GTCACAGAGGCTACTATTAC***TGGCCTGGAACCGGGAAC

5761 ---------+---------+---------+---------+---------+---------+ 5820

CCAGGGAGCCGGGGCGGGACCACAGTGTCTCCGATGATAATGACCGGACCTTGGCCCTTG

V P R P R P G V T E A T I T G L E P G T -

CGAATATACAATTTATGTCATTGCCCTGAAGAATAATCAGAAGAGCGAGCCCCTGATTGG

5821 ---------+---------+---------+---------+---------+---------+ 5880

GCTTATATGTTAAATACAGTAACGGGACTTCTTATTAGTCTTCTCGCTCGGGGACTAACC

E Y T I Y V I A L K N N Q K S E P L I G -

AAGGAAAAAGACAGACGAGCTTCCCCAACTGGTAACCCTTCCACACCCCAATCTTCATGG

5881 ---------+---------+---------+---------+---------+---------+ 5940

TTCCTTTTTCTGTCTGCTCGAAGGGGTTGACCATTGGGAAGGTGTGGGGTTAGAAGTACC

R K K T D E L P Q L V T L P H P N L H G -

ACCAGAGATCTTGGATGTTCCTTCCACAGTTCAAAAGACCCCTTTCGTCACCCACCCTGG

5941 ---------+---------+---------+---------+---------+---------+ 6000

TGGTCTCTAGAACCTACAAGGAAGGTGTCA***AGTTTTCTGGGGAAAGCAGT***GGGTGGGACC

3’ primer yw11 5’

P E I L D V P S T V Q K T P F V T H P G -

5’ primer

GTATGACACTGGAAATGGTATTCAGCTTCCTGGCACTTCTGGTCAGCAACCCA***GTGTTGG***

6001 ---------+---------+---------+---------+---------+---------+ 6060

CATACTGTGACCTTTACCATAAGTCGAAGGACCGTGAAGACCAGTCGTTGGGTCACAACC

Y D T G N G I Q L P G T S G Q Q P S V G -

yw 16 3’

***GCAACAAATGATC***TTTGAGGAACATGGTTTTAGGCGGACCACACCGCCCACAACGGCCAC

6061 ---------+---------+---------+---------+---------+---------+ 6120

CGTTGTTTACTAGAAACTCCTTGTACCAAAATCCGCCTGGTGTGGCGGGTGTTGCCGGTG

Q Q M I F E E H G F R R T T P P T T A T -

CCCCATAAGGCATAGGCCAAGACCATACCCGCCGAATGTAGGACAAGAAGCTCTCTCTCA

6121 ---------+---------+---------+---------+---------+---------+ 6180

GGGGTATTCCGTATCCGGTTCTGGTATGGGCGGCTTACATCCTGTTCTTCGAGAGAGAGT

P I R H R P R P Y P P N V G Q E A L S Q -

GACAACCATCTCATGGGCCCCATTCCAGGACACTTCTGAGTACATCATTTCATGTCATCC

6181 ---------+---------+---------+---------+---------+---------+ 6240

CTGTTGGTAGAGTACCCGGGGTAAGGTCCTGTGAAGACTCATGTAGT***AAAGTACAGTAGG***

3’ primer

T T I S W A P F Q D T S E Y I I S C H P -

TGTTGGCACTGATGAAGAACCCTTACAGTTCAGGGTTCCTGGAACTTCTACCAGTGCCAC

6241 ---------+---------+---------+---------+---------+---------+ 6300

***ACAACCG***TGACTACTTCTTGGGAATGTCAAGTCCCAAGGACCTTGAAGATGGTCACGGTG

yw9 3^#^

V G T D E E P L Q F R V P G T S T S A T -

GG?

5’ primer yw18

TCTGACAGGCCTCACCAGAGGTGCCACCTACAACATCATAGTGG***AGGCACTGAAAGACCA***

6301 ---------+---------+---------+---------+---------+---------+ 6360

AGACTGTCCGGAGTGGTCTCCACGGTGGATGTTGTAGTATCACCTCCGTGACTTTCTGGT

L T G L T R G A T Y N I I V E A L K D Q -

3’

***GCAG***AGGCATAAGGTTCGGGAAGAGGTTGTTACCGTGGGCAACTCTGTCAACGAAGGCTT

6361 ---------+---------+---------+---------+---------+---------+ 6420

CGTCTCCGTATTCCAAGCCCTTCTCCAACAATGGCACCCGTTGAGACAGTTGCTTCCGAA

Q R H K V R E E V V T V G N S V N E G L -

GAACCAACCTACGGATGACTCGTGCTTTGACCCCTACACAGTTTCCCATTATGCCGTTGG

6421 ---------+---------+---------+---------+---------+---------+ 6480

CTTGGTTGGATGCCTACTGAGCACGAAACTGGGGATGTGTCAAAGGGTAATACGGCAACC

N Q P T D D S C F D P Y T V S H Y A V G -

AGATGAGTGGGAACGAATGTCTGAATCAGGCTTTAAACTGTTGTGCCAGTGCTTAGGCTT

6481 ---------+---------+---------+---------+---------+---------+ 6540

TCTACTCACCCTTGCTTACAGACTTAGTCCGAAATT***TGACAACACGGTCACGAATC***CGAA

3’ primer yw7 5’

D E W E R M S E S G F K L L C Q C L G F -

TGGAAGTGGTCATTTCAGATGTGATTCATCTAGATGGTGCCATGACAATGGTGTGAACTA

6541 ---------+---------+---------+---------+---------+---------+ 6600

ACCTTCACCAGTAAAGTCTACACTAAGTAGATCTACCACGGTACTGTTACCACACTTGAT

G S G H F R C D S S R W C H D N G V N Y -

5’ primer yw 20 3’

CAAGATTGGAGAGAAGTGGACCG***TCAGGGAGAAAATGGCCAGA***TGATGAGCTGCACATG

6601 ---------+---------+---------+---------+---------+---------+ 6660

GTTCTAACCTCTCTTCACCCTGGCAGTCCCTCTTTTACCGGTCTACTACTCGACGTGTAC

K I G E K W D R Q G E N G Q M M S C T C -

TCTTGGGAACGGAAAAGGAGAATTCAAGTGTGACCCTCATGAGGCAACGTGTTACGATGA

6661 ---------+---------+---------+---------+---------+---------+ 6720

AGAACCCTTGCCTTTTCCTCTTAAGTTCACACTGGGAGTACTCCGTTGCACAATGCTACT

L G N G K G E F K C D P H E A T C Y D D -

TGGGAAGACATACCACGTAGGAGAACAGTGGCAGAAGGAATATCTCGGTGCCATTTGCTC

6721 ---------+---------+---------+---------+---------+---------+ 6780

ACCCTTCTGTATGGTGCATCCTCTTGTCACCGTCTTCCTTATAGAGCCACGGTAAACGAG

G K T Y H V G E Q W Q K E Y L G A I C S -

CTGCACATGCTTTGGAGGCCAGCGGGGCTGGCGCTGTGACAACTGCCGCAGACCTGGGGG

6781 ---------+---------+---------+---------+---------+---------+ 6840

GACGTGTACGAAACCTCCGGTCGCCCCGACCGCGACACTGTTGACGGCGTCTGGACCCCC

C T C F G G Q R G W R C D N C R R P G G -

TGAACCCAGTCCCGAAGGCACTACTGGCCAGTCCTACAACCAGTATTCTCAGAGATACCA

6841 ---------+---------+---------+---------+---------+---------+ 6900

***ACTTGGGTCAGGGCTTCCGT***GATGACCGGTCAGGATGTTGGTCATAAGAGTCTCTATGGT

3’ primer yw5 5’

E P S P E G T T G Q S Y N Q Y S Q R Y H -

5’ primer yw 22 3’

TCAGAGAACAAACACTAATGTTAATTGCC***CAATTGAGTGCTTCATGCCT***TTAGATGTACA

6901 ---------+---------+---------+---------+---------+---------+ 6960

AGTCTCTTGTTTGTGATTACAATTAACGGGTTAACTCACGAAGTACGGAAATCTACATGT

Q R T N T N V N C P I E C F M P L D V Q -

GGCTGACAGAGAAGATTCCCGAGAGTAAATCATCTTTCCAATCCAGAGGAACAAGCATGT

6961 ---------+---------+---------+---------+---------+---------+ 7020

CCGACTGTCTCTTCTAAGGGCTCTCATTTAGTAGAAAGGTTAGGTCTCCTTGTTCGTACA

A D R E D S R E * I I F P I Q R N K H V -

CTCTCTGCCAAGATCCATCTAAACTGGAGTGATGTTAGCAGACCCAGCTTAGAGTTCTTC

7021 ---------+---------+---------+---------+---------+---------+ 7080

GAGAGACGGTTCTAGGTAGATTTGACCTCACTACAATCGTCTGGGTCGAATCTCAAGAAG

S L P R S I * T G V M L A D P A * S S S -

TTTCTTTCTTAAGCCCTTTGCTCTGGAGGAAGTTCTCCAGCTTCAGCTCAACTCACAGCT

7081 ---------+---------+---------+---------+---------+---------+ 7140

AAAGAAAGAATTCGGGAAACGAGACCTCCTTCAAGAGGTCGAAGTCGAGTTGAGTGTCGA

F F L K P F A L E E V L Q L Q L N S Q L -

5’

TCTCCAAGCATCACCCTGGGAGTTTCCTGAGGGTTTTCTCATAAATGAGGGCTGCACAT***T***

7141 ---------+---------+---------+---------+---------+---------+ 7200

AGAGGTTCGTAGTGGGACCCTCAAAGGACTCCCAAAAGAGTATTTACTCCCGACGTGTAA

L Q A S P W E F P E G F L I N E G C T L -

Primer yw 24 3’

***GCCTGTTCTGCTTCGAAGT***ATTCAATACCGCTCAGTATTTTAAATGAAGTGATTCTAAGA

7201 ---------+---------+---------+---------+---------+---------+ 7260

CGGACAAGACGAAGCTTCATAAGTTATGGCGAGTCATAAAATTTACTTCACTAAGATTCT

P V L L R S I Q Y R S V F * M K * F * D -

TTTGGTTTGGGATCAATAGGAAAGCATATGCAGCCAACCAAGATGCAAATGTTTTGAAAT

7261 ---------+---------+---------+---------+---------+---------+ 7320

AAACCAAACCCTAGTTATCCTTTCGTATACGTCGGTTGGTTCTACGTTT***ACAAAACTTTA***

3’ primer

L V W D Q * E S I C S Q P R C K C F E M -

GATATGACCAAAATTTTAAGTAGGAAAGTCACCCAAACACTTCTGCTTTCACTTAAGTGT

7321 ---------+---------+---------+---------+---------+---------+ 7380

***CTATACTGG***TTTTAAAATTCATCCTTTCAGTGGGTTTGTGAAGACGAAAGTGAATTCACA

yw4 5’

I * P K F * V G K S P K H F C F H L S V -

5’

CTGGCCCGCAATACTGTAGGAACAAGCATGATCTTGTTACTGTGATATTTTAAATAT***CCA***

7381 ---------+---------+---------+---------+---------+---------+ 7440

GACCGGGCGTTATGACATCCTTGTTCGTACTAGAACAATGACACTATAAAATTTATAGGT

W P A I L * E Q A * S C Y C D I L N I H -

primer yw 26 3’

***CAGTACTCACTTTTTCC***AAATGATCCTAGTAATTGCCTAGAAATATCTTTCTCTTACCTG

7441 ---------+---------+---------+---------+---------+---------+ 7500

GTCATGAGTGAAAAAGGTTTACTAGGATCATTAACGGATCTTTATAGAAAGAGAATGGAC

S T H F F Q M I L V I A * K Y L S L T C -

TTATTTATCAATTTTTCCCAGTATTTTTATACGGAAAAAATTGTATTGAAAACACTTAGT

7501 ---------+---------+---------+---------+---------+---------+ 7560

AATAAATAGTTAAAAAGGGTCATAAAAATATGCCTTTTTTAACATAACTTTTGTGAATCA

Y L S I F P S I F I R K K L Y * K H L V -

ATGCAGTTGATAAGAGGAATTTGGTATAATTATGGTGGGTGATTATTTTTTATACTGTAT

7561 ---------+---------+---------+---------+---------+---------+ 7620

TACGTCAACTATTCTCCTTAAACCATATTAATACCACCCACTAATAAAAAATATGACATA

C S * * E E F G I I M V G D Y F L Y C M -

GTGCCAAAGCTTTACTACTGTGGAAAGACAACTGTTTTAATAAAAGATTTACATTCCACA

7621 ---------+---------+---------+---------+---------+---------+ 7680

CACGGTTTCGAAATGATGACACCTTTCTGTTGACAAAATTATTTTCTAAATGTAAGGTGT

C Q S F T T V E R Q L F * * K I Y I P -
