# Supplementary material for: The monoclonal antibody SM5-1 recognizes a fibronectin variant which is widely expressed in melanoma
Source: BMC Cancer. 2006 Jan 11;6:8. doi: 10.1186/1471-2407-6-8 (PMC1351261; doi:10.1186/1471-2407-6-8)
Supplement: Additional File 3 — IIICS region of fibronectin. Human fibronectin gene IIICS region for extra domain (IIICS = type III connecting strand). Genbank Accession NR. X04530. Arrowheads are intron-exon junctions. The 93 bp intron which can be part of an exon and whose presences in our positive clones were different is underlined. a: alternative splicing acceptor site; d: alternative splicing donor site. 1......14 bp: intron. 14......15: intron-exon junction, a1: acceptor site. 15......481: complex fibronectin IIICS exon and coding region containing different splice sites. 89......90: intron-exon junction, a2 acceptor site. 282......283: exon-intron junction, d1 donor site. 282......374: intron sequence or alternatively part of exon by alternate splicing. 374: intron-exon junction, a3 acceptor site. 481......482: exon-intron junction, d2 donor site. 482......1052: intron. In our positive clones, clone 185 containing the alternative part of exon (282......374) and other eight positive clones have the exon from the 15 bp to 481 bp excluding the exon from 282 bp to 374 bp. [file 1471-2407-6-8-S3.ppt]

## Slide 1
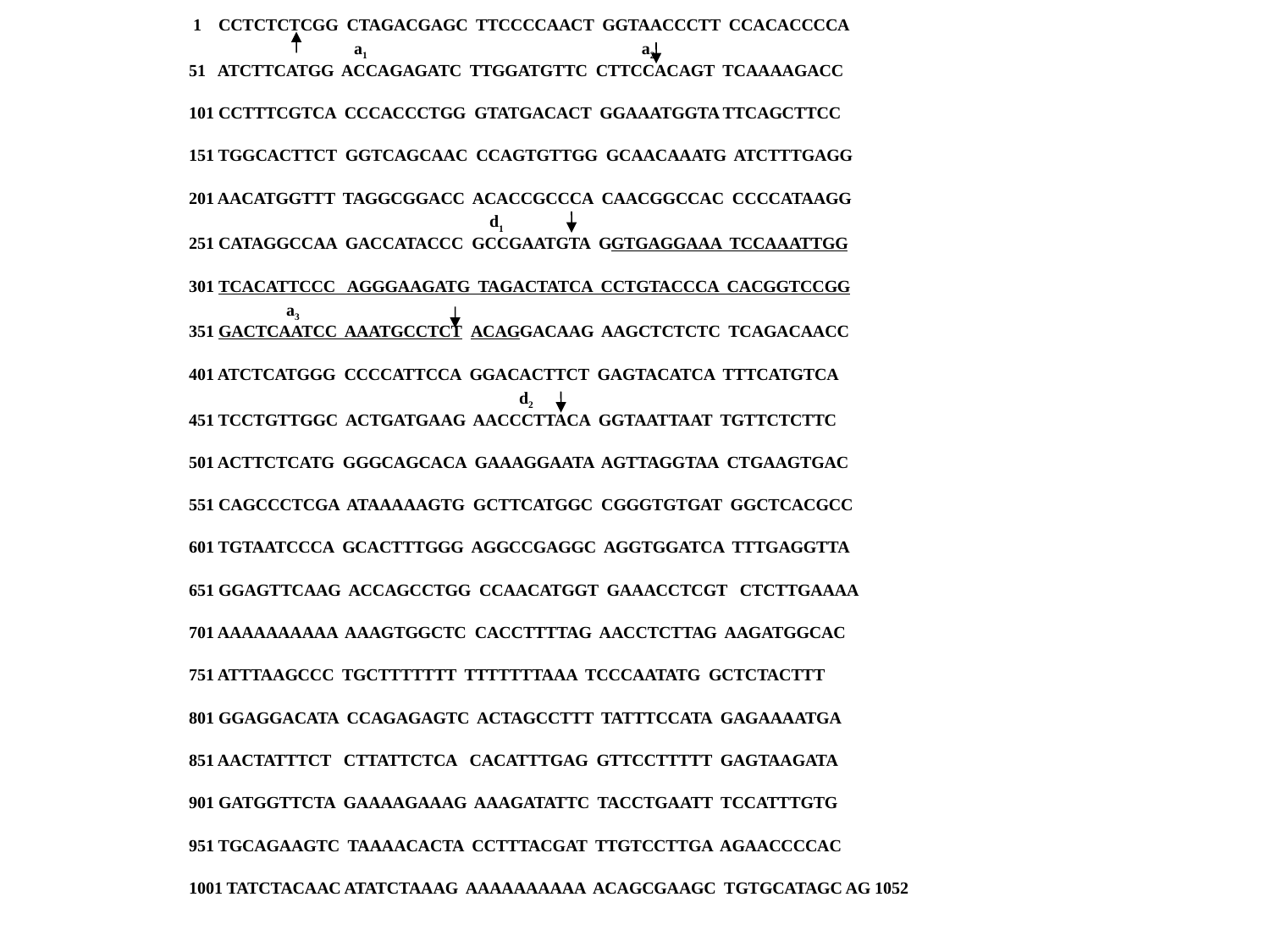

# 1 CCTCTCTCGG CTAGACGAGC TTCCCCAACT GGTAACCCTT CCACACCCCA
 a1 a2
51 ATCTTCATGG ACCAGAGATC TTGGATGTTC CTTCCACAGT TCAAAAGACC
101 CCTTTCGTCA CCCACCCTGG GTATGACACT GGAAATGGTA TTCAGCTTCC
151 TGGCACTTCT GGTCAGCAAC CCAGTGTTGG GCAACAAATG ATCTTTGAGG
201 AACATGGTTT TAGGCGGACC ACACCGCCCA CAACGGCCAC CCCCATAAGG
		 d1
251 CATAGGCCAA GACCATACCC GCCGAATGTA GGTGAGGAAA TCCAAATTGG
301 TCACATTCCC AGGGAAGATG TAGACTATCA CCTGTACCCA CACGGTCCGG
			 a3
351 GACTCAATCC AAATGCCTCT ACAGGACAAG AAGCTCTCTC TCAGACAACC
401 ATCTCATGGG CCCCATTCCA GGACACTTCT GAGTACATCA TTTCATGTCA
	 d2
451 TCCTGTTGGC ACTGATGAAG AACCCTTACA GGTAATTAAT TGTTCTCTTC
501 ACTTCTCATG GGGCAGCACA GAAAGGAATA AGTTAGGTAA CTGAAGTGAC
551 CAGCCCTCGA ATAAAAAGTG GCTTCATGGC CGGGTGTGAT GGCTCACGCC
601 TGTAATCCCA GCACTTTGGG AGGCCGAGGC AGGTGGATCA TTTGAGGTTA
651 GGAGTTCAAG ACCAGCCTGG CCAACATGGT GAAACCTCGT CTCTTGAAAA
701 AAAAAAAAAA AAAGTGGCTC CACCTTTTAG AACCTCTTAG AAGATGGCAC
751 ATTTAAGCCC TGCTTTTTTT TTTTTTTAAA TCCCAATATG GCTCTACTTT
801 GGAGGACATA CCAGAGAGTC ACTAGCCTTT TATTTCCATA GAGAAAATGA
851 AACTATTTCT CTTATTCTCA CACATTTGAG GTTCCTTTTT GAGTAAGATA
901 GATGGTTCTA GAAAAGAAAG AAAGATATTC TACCTGAATT TCCATTTGTG
951 TGCAGAAGTC TAAAACACTA CCTTTACGAT TTGTCCTTGA AGAACCCCAC
1001 TATCTACAAC ATATCTAAAG AAAAAAAAAA ACAGCGAAGC TGTGCATAGC AG 1052
